# Synthesis of Polyether, Poly(Ether Carbonate) and Poly(Ether Ester) Polyols Using Double Metal Cyanide Catalysts Bearing Organophosphorus Complexing Agents

**DOI:** 10.3390/polym16060818

**Published:** 2024-03-14

**Authors:** Eun-Gyeong Lee, Chinh-Hoang Tran, Ju-Yeong Heo, So-Young Kim, Ha-Kyung Choi, Byeong-Ryeol Moon, Il Kim

**Affiliations:** School of Chemical Engineering, Pusan National University, Busandaehag-ro 63-2, Geumjeong-gu, Busan 46241, Republic of Korea; rud6063@pusan.ac.kr (E.-G.L.); chinhtran@pusan.ac.kr (C.-H.T.); hjy0496@pusan.ac.kr (J.-Y.H.); ksy906@pusan.ac.kr (S.-Y.K.); aomghwm0116@pusan.ac.kr (H.-K.C.); moonbro@pusan.ac.kr (B.-R.M.)

**Keywords:** double metal cyanide catalyst, organophosphorus, polycarbonate, polyester, polyether, polyols, ring-opening polymerization, carbon dioxide

## Abstract

We developed a series of Zn(II)-Co(III) double metal cyanide (DMC) catalysts with exceptional activity for the ring-opening polymerization of various cyclic monomers by employing diverse organophosphorus compounds as complexing agents (CAs). The chemical structure and composition of DMC catalysts were investigated by commonly used analysis such as infrared and X-ray photoelectron spectroscopies, and elemental analysis combining with in situ NMR analysis to determine the complexation types of organophosphorus compounds the catalyst framework. The resulting catalysts exhibited very high turnover frequencies (up to 631.4 min^−1^) in the ring-opening polymerization (ROP) of propylene oxide and good efficiency for the ROP of ε-caprolactone. The resultant polyester polyols are suitable to use as an macroinitiator to produce well-defined poly(ester ether) triblock copolymers of 1800–6600 g mol^−1^ and dispersity of 1.16–1.37. Additionally, the DMC catalysts bearing organophosphorus compounds CAs exhibited remarkable selectivity for the copolymerization of PO with CO_2_, yielding poly(ether carbonate) polyols with carbonate contents up to 34.5%. This study contributes to the development of efficient DMC catalytic systems that enable the synthesis of high-quality polyols for various applications.

## 1. Introduction

Double metal cyanide (DMC) complexes, also known as Prussian blue analogs (PBAs), are inorganic coordination compounds consisting of two metal atoms connected through cyanide linkages. The crystal structures of these materials depend on the metals and the preparation method employed. Typically, PBAs with cubic or rhombohedral crystal structures are prepared by mixing aqueous solutions of a metal salt with a metal cyanide salt, without using an organic additive. They are highly crystalline and stable, and have been employed in battery materials, electrochemical sensors, and sorbents [1,2,3,4,5,6,7]. However, these compounds are rarely used as catalysts due to their negligible activities in organic reactions. Since the 1960s, the General Tire and Rubber company has reported highly active PBA compounds, more commonly known as DMC catalysts, for the ring-opening polymerization (ROP) of epoxides to produce high-quality polyalkylene ethers used in the production of polyurethanes [8]. The DMC catalysts produced polyether polyols with lower unsaturation and higher molecular weights than conventional alkaline catalysts. Furthermore, this method eliminates the need for additional purification processes, reduces the number of production steps, and can be employed in continuous processes [9,10,11]. DMC catalysts are prepared in the presence of organic additives or complexing agents (CAs), which result in more amorphous structures than conventional PBAs. To date, DMC catalysts have been widely used for numerous organic reactions such as the copolymerization and cycloaddition of CO_2_ and epoxides, epoxide ring-opening using amines, esterification and transesterification, synthesis of hyperbranched polymers, hydrogen amination reactions, and Prins reactions [12,13,14,15,16,17,18]. Notably, the DMC method has tremendous potential for commercialization of CO_2_ utilization processes. Nevertheless, commonly used DMC catalysts bearing *tert*-butyl alcohol suffer from a long induction period in the ROP of epoxides before the actual propagation can occur. Furthermore, environmental issues caused by the use of excess organic CAs and heavy metal salts, and the complexity of catalyst preparation and characterization render this method less attractive for scientific purposes [19].

To ensure the efficient production of DMC catalysts, proper catalyst selection and coordination with CAs within the catalytic sites are crucial. Since 1996, efforts have been made to develop a more cost-effective and environmentally friendly DMC system that enhances catalyst performance. Strategies to improve DMC catalysts include the replacement of CAs and co-complexing agents, the introduction of different central metals, and the incorporation of various additives. These modifications aimed to broaden the range of DMC catalysts suitable for propylene oxide (PO) ring-opening polymerization and PO/CO_2_ copolymerization [20,21,22].

**Figure 1:**
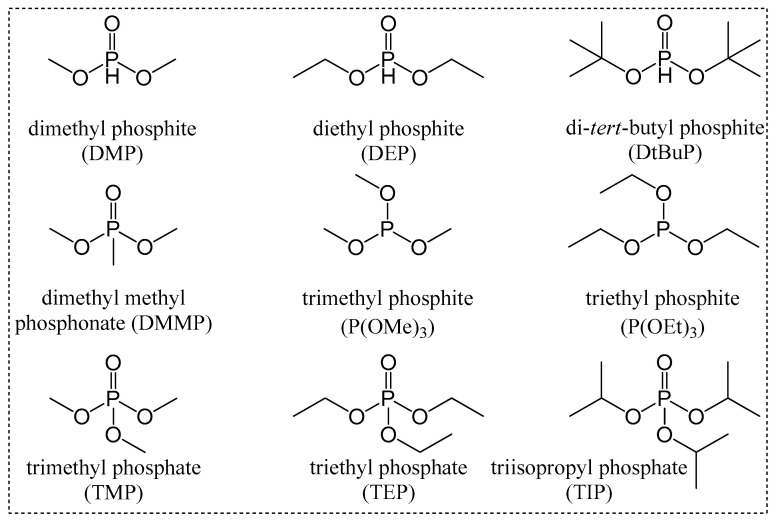
Organophosphorus compounds used as CAs for DMC catalysts.

Phosphoryl groups, such as the P=O moiety can act as Lewis bases and, when combined with Lewis acid metal ions, enable bifunctional activation in various ring-opening polymerization reactions [23]. Additionally, the phosphonate linkers present in various catalysts can exist in three protonatable states, facilitating the formation of effective Brønsted acids for the chemical conversion of CO_2_. Phosphonate materials, which are characterized by increased charges and a higher number of oxygen atoms, are expected to exhibit stronger interactions with metal ions than with carboxylate ligands [24]. In this study, we aimed to expand the variety of DMC catalysts suitable for the ROP of PO and PO/CO_2_ copolymerization. We synthesized a series of zinc/cobalt DMC catalysts using diverse organophosphorus compounds (OPCs) as CAs (Figure 1). The locations and states of the OPCs within the catalyst framework were analyzed using Fourier transform infrared spectroscopy (FTIR) and X-ray photoelectron spectroscopy (XPS). The structures and morphologies of the prepared catalysts were characterized using powder X-ray diffraction (XRD) and high-resolution low-voltage scanning electron microscopy (HRLV-SEM). The chemical compositions of the DMC catalysts were estimated using inductively coupled plasma-optical emission spectroscopy (ICP-OES), thermogravimetric analysis (TGA), and elemental analysis (EA). The resulting polyols were characterized using ^1^H nuclear magnetic resonance (1D-^1^H NMR and ^1^H diffusion-ordered spectroscopy (DOSY) NMR), gel permeation chromatography (GPC), unsaturation level [25], and hydroxyl value using ASTM standards [26].

## 2. Materials and Methods

### 2.1. Materials

Polyethylene glycol (PEG)-*block*-polypropylene glycol (PPG)-*block*-PEG triblock copolymers, Pluronic^®^ F108 (molecular weight (MW) = 14,600 g mol^−1^), P123 (MW = 5800), L-121 (MW = 4400), L-35 (MW = 1900), and L-31 (MW = 1100), were purchased from Merck KGaA (Darmstadt, Germany). ε-Caprolactone (CL), dimethyl phosphite (DMP), di-*tert*-butyl phosphite (DtBuP), dimethyl methyl phosphonate (DMMP), trimethyl phosphite (P(OMe)_3_), triethyl phosphite (P(OEt)_3_), triisopropyl phosphate (TIP), phosphorous acid (H_3_PO_3_) were also purchased from Merck KGaA. Ethylene glycol (EG, >99%) was purchased from Daejung Chemicals and Materials (Dajeon, Republic of Korea). Anhydrous zinc chloride (ZnCl_2_, ≥98%), potassium hexacyanocobaltate (K_3_[Co(CN)_6_], ≥97%), diethyl phosphonate (DEP), trimethyl phosphate (TMP), triethyl phosphate (TEP) were purchased from Thermo Fisher Scientific Korea (Seoul, Republic of Korea). Propylene oxide (PO), PPG-600 (MW = 600 g mol^−1^, functionality (*F*) = 2)) were donated by SK PUCORE (Ulsan, Republic of Korea). Carbon dioxide of 99% (Samjuk Special Gas, Anseong, Republic of Korea) purity was used. All materials were used without additional purification.

### 2.2. Preparation of DMC Catalysts Bearing OPCs

A series of DMC catalysts bearing OPC CAs (DMC-OPCs) were synthesized by reacting aqueous solutions of ZnCl_2_ and K_3_Co(CN)_6_ in the presence of an OPC CA (e.g., DMP, DEP, DtBuP, DMMP, P(OMe)_3_, P(OEt)_3_, TMP, TEP, TIP, and H_3_PO_3_) and a triblock copolymer as co-CA (e.g., F108, P123, L-121, L-35, and L-31). Typically, DMC-TEP catalysts were prepared according to the following procedure. First, solution 1 consisting of ZnCl_2_ (2.05 g, 15 mmol) and TEP (0.1 mL) in 2.5 mL of water was reacted with solution 2 containing K_3_Co(CN)_6_ (0.5 g, 1.5 mmol) in 2.5 mL of water. The reaction took place for 30 min at 30 °C using a magnetic stirrer hot plate equipped with water bath. Subsequently, solution 3, comprising of P123 (0.1 g) and TEP (1 mL), was introduced and the mixture was stirred for an additional 10 min. The resulting suspension was subjected to centrifugation (at ×7700 for 5 min), followed by washing with 20 mL of water and drying under reduced pressure until a constant weight. To determine the optimized condition for preparing DMC-TEP catalyst, the amounts of TEP used in solution 1 and the reaction temperature were respectively modified to 0.5–1 mL and 50–90 °C. Likewise, the optimized conditions for other DMC-OPCs catalysts were determined and results for the optimization are summarized in Supplementary Table S1. For comparative analysis, DMC-pure was prepared without CA and co-CA, employing a Zn/Co ratio of 10:1. Moreover, DMC-*t*BuOH was synthesized using *t*BuOH as a CA, following the experimental procedure outlined in the prior article [27].

### 2.3. General Procedure for the Semi-Batch ROP of PO

The polymerization of PO was performed following a previously reported protocol [28]. In a typical procedure, a prescribed amount of the DMC catalyst and initiator are added to a 500-mL stainless steel reactor (Parr Instrument Company, Moline, IL, USA). The reactor was then evacuated for 60 min to remove any residual water. The polymerization was carried out at 115 °C with continuous addition of PO monomer until a total monomer consumption of 200 g was reached. The catalytic activity was evaluated by monitoring the induction and reaction times, and calculating the turnover frequency (TOF) using the PO consumption (g) and reaction time (min). All of the tests were repeated three times to ensure the reproducibility and consistency of the results.

### 2.4. General Procedure for the Batch ROP of CL

The batch ROP of CL was conducted under nitrogen atmosphere. In a typical procedure, prescribed amount of DMC-DEP catalyst was introduced into a 10 mL round-bottom flask, followed by purging the reactor with nitrogen at 90 °C for 1 h. Subsequently, EG initiator (1 mmol), and ε-CL (10 mmol) was added to the reactor at 160 °C. The progress of monomer conversion over time was assessed by analyzing ^1^H NMR spectra obtained at 30–60 min intervals.

### 2.5. General Procedure for the Batch Copolymerization of PO with CO_2_

All reactions were carried out in a 160-mL stainless-steel autoclave equipped with a mechanical stirrer, temperature controller, and pressure indicator (Parr Instrument Company, Moline, IL, USA). In a typical procedure, prescribed amount of DMC catalyst and PPG-600 initiator were added into the autoclave and CO_2_ was flowed for 30 min to remove trace of water. Subsequently, a specified amount of monomer (0.35 mmol) and toluene (10 mL) were added and the CO_2_ pressure was raised to approximately 5–30 bar. The reaction was then conducted at 105 °C for 3 h.

### 2.6. Characterization

FTIR spectra were acquired using a Shimadzu-IR Prestige 21 spectrometer (Shimadzu Scientific Korea, Seoul, Republic of Korea). To prepare the solid samples, KBr powder was mixed with the sample and pressed using a mechanical press to form pellets. Spectra were collected in the range of 400–4000 cm^−1^. To conduct elemental analysis of the metal and phosphorus materials (Zn, Co, P), ICP-OES was employed. For element analysis of carbon, hydrogen, and nitrogen, Vario-Micro Cube Elemental Analyzer (EA Korea, Hanam, Republic of Korea) was used. The Model Optima™ 8300—ICP-OES Spectrometer from Perkin Elmer (Hopkinton, MA, USA) was utilized for both of these analyses. We carried out TGA using a TA Instruments-Q500 instrument (TA Instruments Korea, Seoul, Republic of Korea), with a heating rate of 10 min^−1^ under N_2_ atmosphere, according to the ASTM D274-5 standard [29]. For XPS analysis, an ESCALAB 250 X-ray Photoelectron Spectrometer (Thermo Fisher Scientific Korea, Seoul, Republic of Korea) was utilized, with A1 K𝛼 radiation (h𝑣 = 1486.5 eV) emitted from an X-ray source that was operated at 12 mA and 20 kV. We applied a thin layer of Pt on the microstructures of the samples and examined them using a JSM-7900F JSM-7900F Schottky Field Emission Scanning Electron microscope (Jeol Korea, Seoul, Republic of Korea). XRD analysis was conducted using a Rigaku RINT2000 wide angle goniometer 185 (Rigaku Corporation, Tokyo, Japan) with Cu Ka radiation. Polyols were characterized using ^1^H and DOSY NMR and GPC. The Varian INOVA 400 NMR spectrometer (Varian Technology Korea, Seoul, Republic of Korea) and AVANCE NEO 600 (Bruker, Seoul, Republic of Korea) were used to record the ^1^H NMR (400 MHz) and ^1^H 2D DOSY spectra, respectively. GPC was employed to determine the number-average molecular weight (MW) and polydispersity index (*Ð*) of the samples. Low PDI polystyrene standards were used for calibration, and a Waters 150 instrument (Waters Korea, Seoul, Republic of Korea) equipped with 104, 103, and 500 Å columns was used in tetrahydrofuran (THF) at 40 °C. The degree of unsaturation and hydroxyl values were determined using titration (ASTM D4671-05 [25] and E1899-97 [26], respectively). The analytical software used for data analysis included MestReNova version: 6.0.2-5475, SigmaPlot version: 10.0.0.54, and OriginPro 2021 version: 9.8.0.200 with Gaussian function.

## 3. Results and Discussion

### 3.1. Preparation and Characterization of DMC Catalysts

The commonly employed procedures for synthesizing DMC catalysts involves several steps, such as iterative reslurrying and washing of the catalyst suspension with organic CAs and water [28]. These processes aim to enhance the coordination between the CAs and metal sites in the catalyst. However, despite their effectiveness, these methods tend to increase processing costs due to the multiple steps involved, as well as environmental concerns related to the use of large amounts of organic CAs and the generation of waste from the washing steps. To address the existing challenges and gain insight into how different factors such as preparation temperature, the type and quantity of CAs used, and the incorporation of co-CAs influence the effectiveness of the catalyst preparation process, in this study, various organophosphorus compounds such as DMP, DEP, DtBuP, DMMP, P(OMe)_3_, P(OEt)_3_, TMP, TEP, TIP, and H_3_PO_3_ were systematically explored as CAs for synthesizing efficient DMC catalysts.

**Figure 2:**
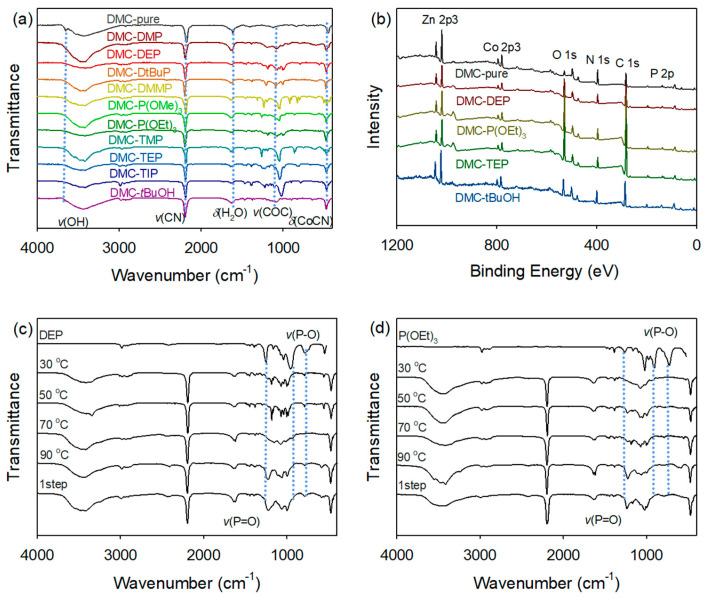
Characterization of various DMC-OPCs catalysts using FTIR (**a**) and XPS (**b**) spectra. FTIR spectra of DMC-DEP (**c**) and DMC-P(OEt)_3_ (**d**) catalysts prepared at different temperatures.

FTIR spectroscopy was used to identify the formation of DMC complexes with OPCs. (Figure 2). The IR spectra of the prepared DMC catalysts display upward shifts in the characteristic C≡N stretching vibration signal, *ν*(C≡N), originally observed at 2080 cm^−1^ in free CN in aqueous solution. These shifts of *ν*(C≡N) from DMC-pure (2177 cm^−1^) to the higher frequencies in the range of 2186–2199 cm^−1^ were attributed to the alteration in the oxidation state of the metal coordination. The increase in the Co–CN bending vibrations of DMC-OPCs catalysts from 450 cm^−1^ to the range of 468–475 cm^−1^ also indicates a change in the coordination environment of the Co centers within the catalyst structure. For all DMC catalysts bearing OPC CAs, the absorbed water signals of *δ*(H−O−H) observed at frequencies ranging from 1616 to 1620 cm^−1^, while the *ν*(C−O−C) bands associated with the P123 appeared at approximately 1080 cm^−1^. The signals assigned to the stretching vibration of P=O in the range of 1198–1259 cm^−1^ exhibit slight red shifts compared to those observed for the pristine CAs. The red shifts, ranging from 14 to 42 cm^−1^, and the absence of a Zn−OH stretching vibration signal suggest neutral coordination between the OPCs and Zn sites during complexation. In contrast, in the case of DMC-pure, where the Zn sites were occupied by chemisorbed water molecules, a sharp peak corresponding to the Zn−OH stretching vibration was detected at 3650 cm^–1^. This peak provides evidence for the presence of lattice water molecules interacting with the Zn sites.

Furthermore, the temperature at which the catalysts were prepared significantly affected the degree of shift in the phosphoryl peak associated with the organophosphite and organophosphonate groups. The FTIR analysis results (Figure 2c,d) indicate that DMC-P(OEt)_3_ catalyst synthesized at temperatures above 50 °C exhibits a distinct *ν*(P=O) signal, which is not observed in trivalent organophosphites. Additionally, DMC-DEP shows a similar FTIR data signal to that of DMC-P(OEt)_3_ at 70 °C. In contrast to phosphines, which are prone to the oxidation and activation of the P−C bond, phosphites and similar compounds containing P−O bonds exhibit inherent instability towards hydrolysis. This instability becomes apparent during the reaction between CA and water during catalyst synthesis. Consequently, the hydrolysis reaction leads to the transformation of the trivalent species into pentavalent species [30,31,32]. In the DMC-TEP catalyst, the influence of temperature was found to be negligible, while only variations in the amount of CA showed discernible differences in the FTIR data. Additional details and results of the FTIR analysis are shown in Supplementary Figures S1–S5 and Tables S3 and S4.

The surface chemical properties of the DMC catalysts were investigated using XPS (Figure 2b). The obtained spectra were calibrated to the C 1s core level peak at 285.0 eV, which corresponds to adventitious carbon on the catalyst surface. The observed peaks were located at 1021.9–1042.6 eV (Zn 2p3), 781–783.3 eV (Co 2p3), 531.2–533.5 eV (O 1s), 398.2–399.6 eV (N 1s), 285–286.6 eV (C 1s), 198.5–199.7 eV (Cl 2p), and 133.9–134.9 eV (P 2p). The Zn 2p_3/2_ spin orbital binding energy of the DMC catalysts exhibited a slight decrease compared to that of free ZnCl_2_, which can be attributed to the presence of CN ligands, inducing a relatively higher charge density on the Zn atoms. Additionally, the binding energy of zinc atoms in the DMC catalysts bearing OPCs showed a slight increase compared to DMC-pure, indicating the coordination of complexing agents to the metal sites, resulting in a shift of approximately 2 eV. These findings are consistent with the results obtained from FTIR analysis, providing further confirmation of the interaction between the organic CAs and DMC frameworks.

**Figure 3:**
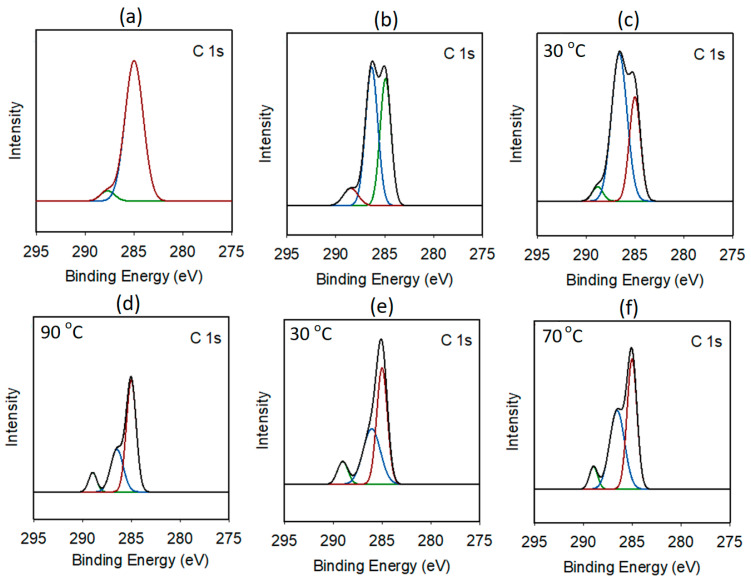
Expanded C 1s XPS spectra: (**a**) DMC-pure, (**b**) DMC-DMMP, (**c**,**d**) DMC-DEP prepared at 30 and 90 °C, respectively, and (**e**,**f**) DMC-P(OEt)_3_ prepared at 30 and 70 °C, respectively.

The deconvolution of the C 1s region of the DMC-pure catalyst revealed a dominant signal at 285 eV, corresponding to the C−C/C−H bonds. In addition, a smaller signal (287.7 eV) was observed, indicating the presence of C−N bonds. In contrast, for the other DMC catalysts, the C 1s spin orbitals were clearly deconvoluted into three distinct signals. The first peak, located at approximately 285 eV, was attributed to the C−H and C−C bonds of co-CA. The second signal at 286.5 eV was assigned to the C−O bonds of co-CA and CA. Finally, a relatively small peak was observed at approximately 288 eV, corresponding to C≡N bonds (Figure 3) [33]. The C 1s regions of the DMC-DEP and DMC-P(OEt)_3_ catalysts exhibited a trend consistent with the FT-IR data. As the catalyst preparation temperature increased, the DMC-DEP catalyst exhibited a decrease in the intensity of the C−O signal and a significant decrease in the binding energy of the P 2p peak. On the other hand, the DMC-P(OEt)_3_ catalyst shows a decrease in the binding energy of the C 1s peak from 286.0 eV to 286.5 eV at 30 °C and 70 °C, respectively. These observations indicate that the catalysts undergo structural changes with temperature variation, which can affect their catalytic performance (Supplementary Table S5). These findings indicate that the coordination environment around the metal site changes in response to temperature-dependent variations in CA. The expended spectra of the Zn 2p3 and Co 2p3 region of DMC-pure, DMC-DMMP, DMC-DEP, and DMC-P(OEt)_3_ are also given in Supplementary Figures S6 and S7. In most case, the Zn 2p3 and Co 2p3 spin orbitals were deconvoluted into two singlet peaks, except for the Zn 2p3 spin orbits of DMC-DEP and DMC-P(OEt)_3_.

The XRD analysis revealed the morphologies of the DMC-OPC catalysts (Supplementary Figure S8). The pure DMC compound exhibited a highly crystalline cubic lattice structure with reflections at 2θ (14.9°, 17.1°, 24.3°, 35.1°, 39.1°, 43.1°). In contrast, the diffraction patterns of the DMC catalysts incorporating organic CAs displayed considerably broadened signals, and some signals at specific d-spacings vanished, suggesting a more amorphous structure and smaller crystalline size. Crystallite sizes of DMC-DEP, P(OEt)_3_, and TEP were calculated from XRD data, resulting in crystal sizes of 11.0 nm, 43.7 nm and 36.7 nm, respectively. Although these structures were not fully amorphous, as evidenced by the sharp reflection peak at 2θ = 23.7° and broad peaks at specific d-spacings, they exhibited the coexistence of cubic, monoclinic, and rhombohedral structures. As the amount of CA increased, the DMC-TEP catalyst underwent a notable transformation from a cubic to a monoclinic structure. This observation indicates that a higher TEP content, such as CA, has a pronounced impact on the morphology of DMC, resulting in a decrease in the cubic crystal structure (Supplementary Figures S9 and S10). The SEM images presented in this study reveal the amorphous morphology of the DMC-DEP catalyst, with the presence of small particles with an average size smaller than 200 nm (Figure S11). These results suggest that the introduction of organic CAs causes changes in the crystal structure and size of the DMC catalysts.

The compositions of the resulting DMC catalysts were determined by ICP-OES, elemental analysis, and TGA. The Zn, Co, and P contents of these catalysts were estimated using ICP-OES and were found to range from 16.9% to 27.6%, 8.3% to 16.6%, and 3.5% to 6.9%, respectively. Additionally, the organic component content was determined using elemental analysis and TGA. The catalysts exhibited higher Zn/Co content compared to the DMC-pure. The TGA curves of the DMC catalysts are depicted in Supplementary Figure S12. The TGA results demonstrated a substantial reduction in matrix water content, decreasing from 12 wt% in the pure DMC to 0.5–16.3 wt% in the DMC catalysts incorporating various OPC CAs. This decrease was attributed to the replacement of water molecules within the DMC framework with CA molecules during the preparation stage. In the case of the pure DMC catalyst, weight loss acceleration was observed up to approximately 200 °C. After this temperature, the derivative weight exhibited a declining behavior until around 560 °C when the Zn-Co structure started to decompose. For the DMC-*t*BuOH catalyst, after the stage at 230 °C, there was another increase in dm/dT, and the sample weight decreased by 13.0% upon reaching a temperature of 400 °C. The peculiar decomposition behavior of the DMC-*t*BuOH catalyst was attributed to the presence of co-CA. The TGA curves also indicated differences in decomposition behavior of these catalysts at temperatures higher than 100 °C, implying distinct organization of Zn-Co cyanide structures in these materials. The percentage of CAs incorporated into the catalyst structures ranged from 10.2% to 21.7%, depending on the specific CAs used. The estimated formulae for the resulting catalysts are listed in Table 1 [34].

### 3.2. Catalytic Activities of DMC Catalysts

**Figure 4:**
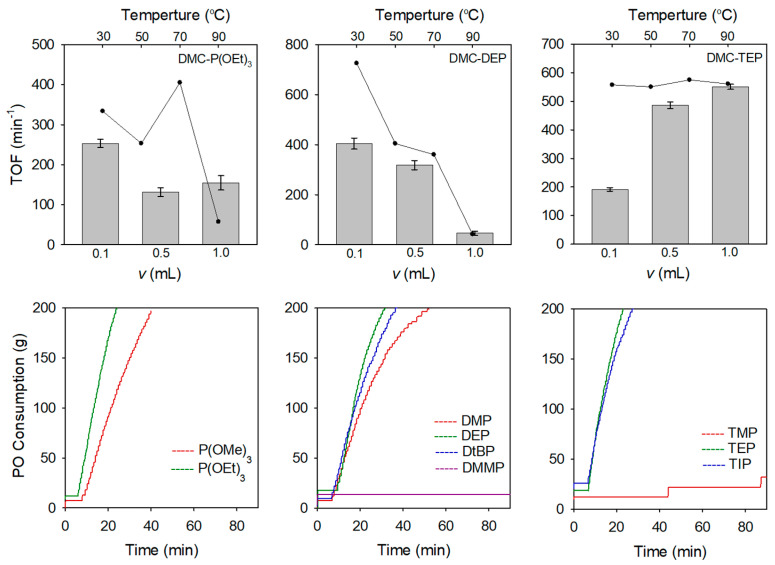
Optimization of DMC-OPCs catalysts for the ROP of PO and PO polymerization rate curves of DMC-OPCs obtained by various type of OPCs.

A series of DMC catalysts incorporating different OPC CAs were initially investigated for the ROP of PO. Various initiator systems were employed during the reactions, and the consumption of PO was monitored over time. The optimal conditions for the DMC catalyst preparation were determined by adjusting the amount of CA and the reaction temperature. Figure 4 presents a comprehensive overview of the turnover frequency (TOF) values, varying amounts of CA and temperatures, and the PO polymerization curves obtained for different types of CA. The DMC-P(OEt)_3_, DMC-DEP, and DMC-TEP catalysts were prepared by conducting experiments with different amounts of CA and reaction temperatures to optimize the synthesis process. The manufacturing conditions for each catalyst are listed in Supplementary Table S1.

At 50 °C, DMC-P(OEt)_3_ and DMC-DEP catalysts showed the highest activities (TOFs of 253 min^−1^ and 404 min^−1^, respectively) when using the lowest amount of CA whereas DMC-TEP exhibited the highest activity when the highest amount of CA was used (TOF = 550 min^−1^) (Supplementary Figures S13–S15). Specifically, the DMC-DEP and DMC-P(OEt)_3_ catalysts exhibited distinct TOF trends depending on the reaction temperature. According to the analysis of the catalytic activity of the DMC-P(OEt)_3_ catalyst at different synthesis temperatures, it was observed that the catalyst synthesized at 70 °C, which showed a similar FTIR spectrum to that of the DMC-DEP catalyst, displayed the highest catalytic activity. This observation indicates that the presence of an oxygen-rich pentavalent form contributes to the enhanced catalytic activity. In contrast, the activity of the DMC-DEP catalyst significantly decreased with increasing temperature.

**Figure 5:**
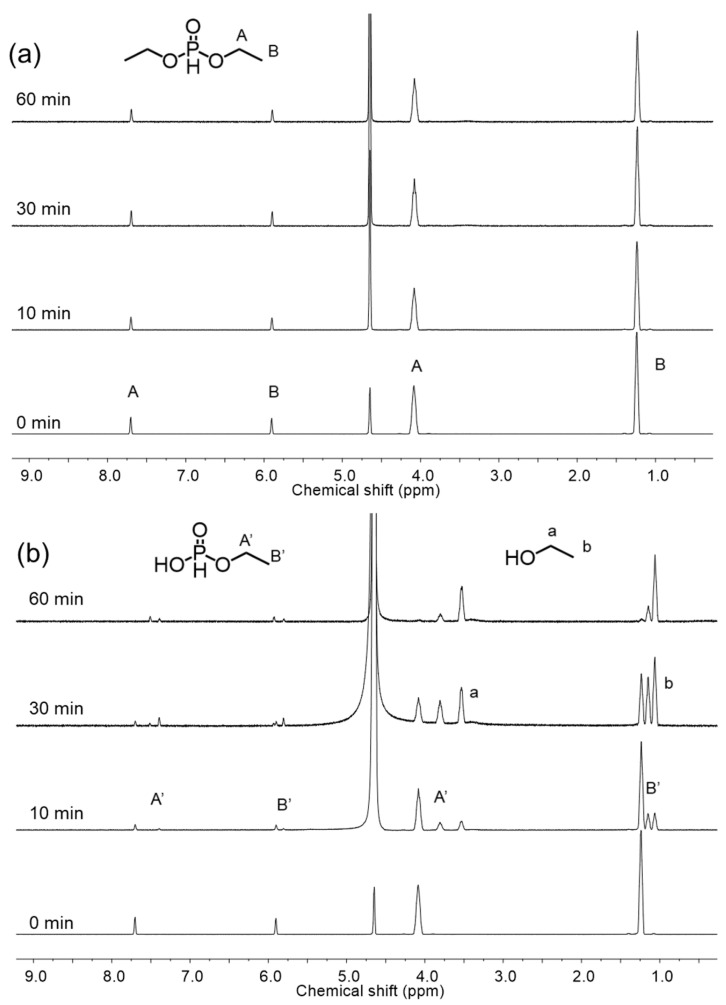
Variable-time ^1^H NMR spectra of the hydrolysis of DEP in D_2_O at (**a**) 30 °C and (**b**) 90 °C. Conditions were the same as those used in the preparation of DMC-DEP.

For the characterization of DMC catalysts bearing organophosphorus compounds, it is expected to cope with difficulties to determine the nature of the active sites since the hydrolysis reaction of these compounds leads to the formation of the multivalent phosphorus derivatives. Therefore, in situ NMR analysis was investigated to understand the transformation behavior of organophosphorus compounds during catalyst preparation. To investigate the impact of the preparation temperature on the hydrolysis of OPC, in situ ^1^H NMR spectra were collected during preparation of DMC-DEP using D_2_O at 30 °C and 90 °C (Figure 5). The experimental results indicate that hydrolysis did not occur during catalyst preparation at 30 °C, but a rapid hydrolysis reaction was observed at 90 °C. It is determined that the catalyst was converted to H_3_PO_3_ form at 90 °C. The FTIR spectra of the catalyst prepared using H_3_PO_3_ as the final hydrolysis product of the organophosphonate revealed weak coordination between the CA and Zn sites, which corresponded to a decrease in the activity towards PO. In contrast, TEP exhibited similar reactivity regardless of temperature (Supplementary Figure S15). Additionally, hydrolytic stability varied depending on the specific substituents present [35]. The PO polymerization curves presented in Figure 4 illustrates the comparison of polymerization activities of PO at 70 °C using catalysts prepared with various OPCs having different substituents (Supplementary Table S6). In all DMC catalysts, a decrease in activity was observed when using CA with methoxy groups. This could be attributed to the lower hydrolytic stability of the alkoxy groups, which led to a higher rate of hydrolysis [36]. In contrast, the DMC-DtBuP catalyst demonstrated exceptional resistance to hydrolysis, primarily due to the steric hindrance provided by the tert-butyl group. However, despite its enhanced hydrolytic stability, the catalytic activity was lower than that of DMC-DEP. As the bulkiness of the substituent at the Zn site increased, the catalytic activity diminished. This decrease in activity can be attributed to the presence of bulky groups resulting from partial hydrolysis, which obstruct the active sites of the catalyst and limit its efficiency [37,38,39]. The results indicate that the type of substituent, particularly the alkoxy group, influences the catalyst activity. Another reason for the decreased activity is the closely related characteristics of the substituents attached to the phosphorus atom and the basicity of oxygen in the phosphoryl group, as demonstrated in other experiments [40].

The DMC catalysts prepared with DMMP under various conditions showed no activity for the ROP of PO, suggesting that DMMP may not effectively interact with the metal centers in the catalyst under the conditions tested. By comparing the DMC-DMMP and DMC-DEP catalysts, an important distinction can be made based on the presence of a methyl group or a hydrogen atom near the P atom. Significant shifts in the FTIR spectra were observed for the R group during catalyst synthesis. The FTIR shifts indicate changes in the coordination environment of the catalyst, whereas the differences in the P=O shift suggest varying binding strengths between the phosphoryl group of CA and the metal matrix. This implied that the DMC-OPC catalysts exhibited different coordination modes of the phosphoryl group with the metal matrix, which were influenced by the basicity of the functional group. Strong coordination of the complexing species to dormant sites impedes rapid exchange reactions with the initiator molecule, leading to a delay in the formation of polymerization centers. This phenomenon hindered the efficient initiation of polymerization and delayed the overall polymerization process [9]. Consequently, as the basicity of the phosphoryl group increases, the resulting catalyst binds excessively to both the CA and metal sites, resulting in reduced activity. In the case of organophosphates, it is expected that there will be little difference in reactivity between DMC-TEP and DMC-TIP, indicating that the affinity difference of the phosphoryl group is not significant [41].

To investigate the impact of co-complexing agents (co-CAs) on DMC, active organophosphorus compounds P(OEt)_3_, DEP, and TEP were prepared with and without co-CAs. In a typical experimental procedure, an aqueous solution containing ZnCl_2_ (2.05 g, 15 mmol), K_3_Co(CN)_6_ (0.5 g, 1.5 mmol), CA, and a co-CA above its critical micelle concentration were combined and mixed at an optimized temperature for 30 min. Subsequently, the resultant solids were isolated and washed with water to remove KCl and unreacted substrates. After vacuum drying at 85 °C for 8 h, the catalyst cakes were ground into a fine powder and subjected to catalytic testing. Both DMC-P(OEt)_3_ and DMC-DEP exhibited significantly reduced activity towards PO, with TOF values of 32 and 183 min^−1^, respectively, indicating a noticeable decrease in activity. Interestingly, the catalysts prepared at 90 °C without co-CAs showed similar FTIR data to those with co-CAs (Supplementary Figures S2–S4). The use of co-CAs aims to enhance the activity of DMC by removing water, which is a competitor to the organic CAs. When co-CAs are not used, the presence of water during the synthesis affects hydrolysis, potentially affecting the activity of the catalysts [16].

**Figure 6:**
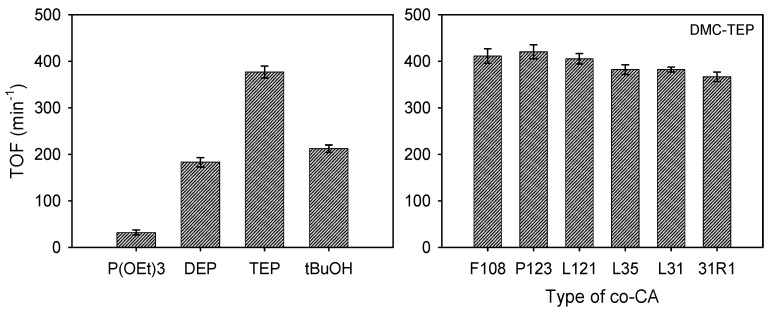
Turnover Frequency (min^−1^) of PO ring-opening polymerization: Comparison between DMC catalyst without co-CA and DMC-TEP catalyst prepared with different Types of co-CA.

On the other hand, DMC-TEP exhibited a high TOF of 377 min^−1^ even without the use of co-CAs. To further investigate this behavior, a series of catalysts was prepared using co-CAs based on PEO-PPO structures with various architectures, and their PO activities were examined (Supplementary Table S2). These results demonstrated a similar trend to the observed PO activity, which was not influenced by temperature, indicating the stability of the catalysts in water (Figure 6 and Supplementary Figure S16). The catalysts bearing organophosphorus CAs, especially DMC-DEP and DMC-TEP, exhibited substantially higher activities towards the ROP of PO despite using much lower amounts of CA compared to DMC-*t*BuOH catalysts. DMC-TEP also exhibited remarkably higher performance toward ROP of PO compared to DMC catalysts using other CAs (e.g., diketones, ketoester, diesters etc.) previously reported by our group even without the use of co-CAs [38]. These results highlight the importance and effectiveness of our procedure for the synthesis of DMC catalysts. The results of the ROP of PO are summarized in Supplementary Table S6. The resultant PPO polyols had moderate *M*_n_ values of 3200–5600 g mol^−1^ and a narrow density of 1.12–1.17. In particular, these polyols were characterized by relatively low unsaturation levels (0.006–0.009), which distinguishes these heterogenous DMC catalysts from conventional alkaline catalysts (e.g., KOH). Additionally, the DMC-DEP catalyst demonstrated remarkable activity for the ROP of CL, as shown in Figure S42.

### 3.3. Copolymerization of PO and CO_2_ Using DMC-OPCs Catalysts

**Figure 7:**
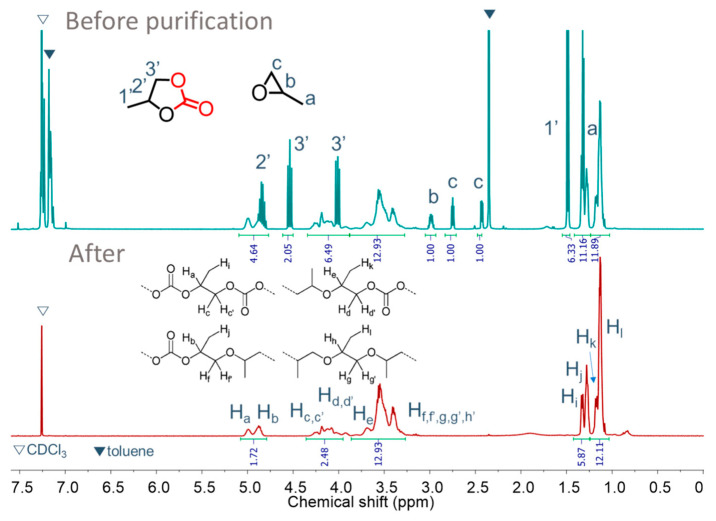
^1^H-NMR spectra of polyether carbonate polyols before and after purification. Reaction condition: Catalyst amount = 50 mg, PO = 0.34 mol, PPG-600 = 0.25 mmol, toluene = 10 mL, PCO_2_ = 30 bar, *T_P_* = 105 °C, *t_P_* = 3 h.

Poly(propylene carbonate) (PPC) polyols were synthesized by copolymerizing PO and CO_2_ in the presence of PPG-600, using DMC-OPCs catalysts. The main products of the copolymerization were PPC polyols, but there was also a formation of cyclic carbonate through backbiting. The optimal reaction conditions, based on carbon dioxide content, MW, and yield, were set, except for the DMC catalysts. The ^1^H NMR spectra of the PPC polyol obtained by DMC-OPCs catalysts are given in Supplementary Figures S25–S41. These spectra were obtained before and after the removal of the cyclic carbonate, residual monomer, and solvent. Analysis of the ^1^H-NMR spectra revealed several distinct signals corresponding to the different functional groups present in the PPC polyol. The signals observed at 4.8–5.0 ppm and 3.9–4.3 ppm were assigned to the CH and CH_2_ groups, respectively, of the carbonate units in the PPC polyol. Peaks in the range of 1.0–1.2 ppm and 1.2–1.4 ppm were attributed to the hydrogen of CH_3_ in the carbonate and ether linkages, respectively. Signals in the range of 3.2–3.8 ppm corresponded to the hydrogen of both CH and CH_2_ groups in the ether linkages. Peaks at 4.85, 4.55, 4.02, and 1.50 ppm confirmed the presence of cyclic carbonate (cPC). In addition, peaks corresponding to unreacted propylene oxide (PO) peaks were detected at 2.4–3.0 ppm (Figure 7).

As shown in Table 2, the DMC catalysts exhibited good to excellent activity for the PO/CO_2_ copolymerization. Further studies were conducted using the DMC-OPC catalysts to explore the potential of controlling the molecular weight and CO_2_ content of the product by adjusting the initiator amount. Increasing the initiator amount led to a decrease in the CO_2_ content but also resulted in a more uniform distribution of the polyol, as evidenced by the observed uniformity when reducing the initiator amount. The optimized DMC-OPC catalyst exhibited a high carbonate content but also yielded a significant amount of byproducts. Experimental observations revealed that the quantities of these byproducts could be regulated by adjusting the CO_2_ pressure.

### 3.4. Synthesis of Triblock Copolymers Using Macroinitiators

**Figure 8:**
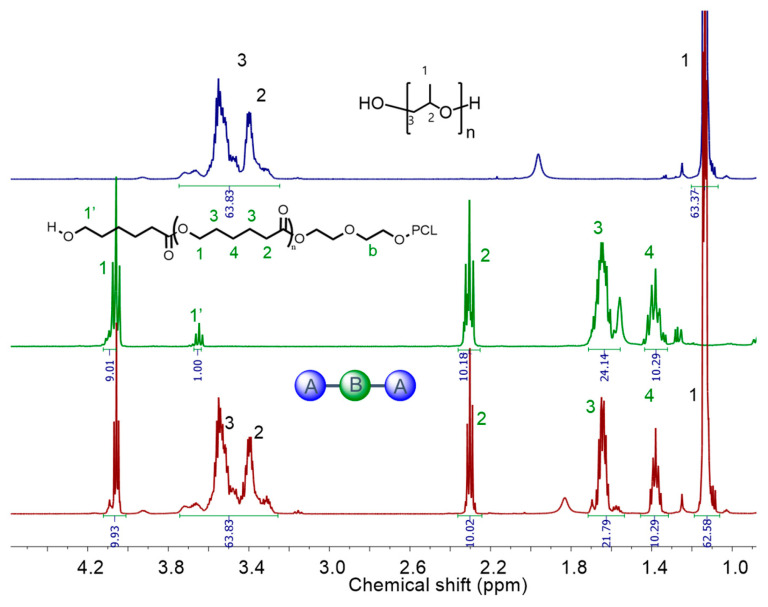
^1^H NMR spectra (400 MHz, CDCl_3_) of PPG, PCL and PPG-PCL-PPG block copolymer (BCP-1). A: Polypropylene glycol (PPG), B: Polycaprolactone (PCL).

**Figure 9:**
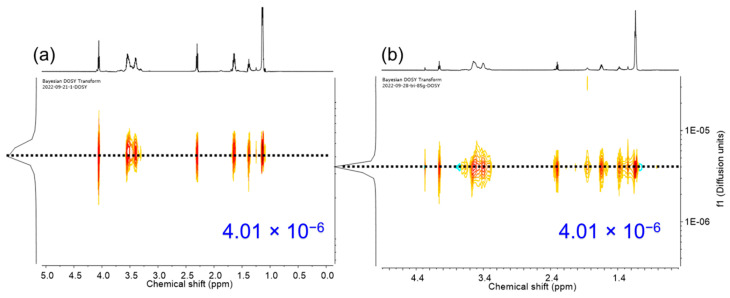
2D DOSY NMR Spectra (600 MHz, CDCl_3_) of (**a**) BCP-1 and (**b**) BCP-2.

The synthesis of PPG-PCL-PPG and PPG-PTMG-PPG triblock copolymers were conducted in a 500-mL stainless-steel reactor using PCLs and PTMGs (*F* = 2, MW = 1400–2000 g mol^−1^) as macroinitiators and DMC-DEP catalyst. The α,ω-hydroxyl-functionalized PCLs were obtained by ROP of CL using EG initiator and DMC-DEP catalyst (Supplementary Figure S42). The resultant copolymers were intensively characterized by both ^1^H and DOSY NMR analysis (Figure 8 and Figure 9 and Supplementary Figures S43–S45).

DOSY NMR spectroscopy is a valuable technique for providing evidence for block copolymer formation. In a successful block copolymerization, where monomer A is polymerized using poly B as an initiator to form poly A-*block*-poly B, the correlation spots representing the ^1^H NMR signals of the poly A and poly B blocks exhibited identical diffusion coefficients [42]. This observation confirms the presence of well-defined block copolymer structures. To further investigate the block copolymers, DOSY NMR experiments were performed on all series of block copolymers (BCPs) in CDCl_3_. The ^1^H NMR spectrum reveals signals attributed to the PCL block (2.30 ppm), the PTMG block (1.62 and 3.41 ppm of methylene), and the PPG block (3.55, 3.40 ppm of methylene, and 1.14 ppm of methyl). The DOSY data clearly illustrated that when PTMG and PPG were present in separate chains, they exhibited distinct diffusion coefficients (Supplementary Figure S45). This observation provides strong evidence for the independent diffusion behaviors of PTMG and PPG, indicating their presence as separate entities within the polymer structure. These results provide crucial insights into the block copolymerization process and confirm the successful formation of well-defined block copolymers consisting of the PTMG and PPG segments. Interestingly, the DOSY map revealed that the ^1^H NMR signals from BCP-1 to BCP-5 exhibited identical diffusion coefficients, with values of 4.01 × 10^−6^ m^2^ s^−1^ and 4.20 × 10^−6^ m^2^ s^−1^, respectively. This consistency indicates efficient polymerization of the epoxide on the PCL and PTMG macroinitiators, supporting the successful formation of block copolymers. As observed from the GPC curves, the resultant block copolymers exhibit relatively narrow dispersity with MW ranging 1800–6600 g mol^−1^ (Supplementary Figure S46). Results for the synthesis of block copolymers are given in Table 3.

## 4. Conclusions

In this study, a series of DMC catalysts were synthesized using different organophosphorus compounds as CAs. The state and coordination types between the OPCs and metal sites within the DMC framework were identified by the changes in characteristic vibrations and signals observed from FTIR and XPS spectra of the catalysts. The interaction between these OPCs and metal sites exhibited a great impact on the crystal structure, crystallite size, and morphology of the resultant DMC catalysts, as evidenced by XRD and SEM analysis. The effects of preparation temperature, type and amount of OPCs, and the type of co-complexing agents on the activity of the catalysts were systematically investigated for the polymerization of PO. The DMC catalysts using OPC CAs demonstrated remarkably higher activity compared to conventional DMC-*t*BuOH catalysts, despite using much lower amounts of CA. Particularly, DMC-TEP catalyst exhibited remarkable activity even without the use of co-CAs. DMC-DEP catalysts exhibited excellent activities with TOF up to 725 min^−1^. DMC catalysts bearing OPC CAs also produced polyols of moderate MW (4100–5600 g mol^−1^), narrow density (1.12–1.17), and particularly low degree of unsaturation (0.006–0.009 meq g^−1^). Additionally, the DMC-DEP catalyst demonstrates remarkable activity for the batch polymerization of CL. The resultant PCLs were employed as α,ω-hydroxyl-functionalized macroinitiators, produced PPG-PCL-PPG and PPG-PTMG-PPG triblock copolymers of controlled MW of 1800–6600 g mol^−1^ and narrow density (1.16–1.37). The DMC catalysts using OPCs also demonstrated good to excellent activity for the copolymerization of PO with CO_2_, resulting in the formation of PPC with high yields (57–76.5%) and carbonate content (20–37%). The polyether, polyester, and poly(ether carbonate) polyols, and triblock copolymers produced by DMC catalysts are potential precursors for the preparation of sustainable and biodegradable thermoplastic polyester and polyurethane elastomers, contributing to the development of efficient and sustainable DMC catalysis.

## Figures and Tables

**Table 1:** Elemental analysis of the DMC catalysts.

Catalyst	ICP-Mass (wt%)			Elemental Analysis (wt%)			TGA (wt%)			Estimated Catalyst Formulation
	Zn	Co	P	C	H	N	CA	H_2_O	P123	
DMC-pure	27.6	16.6	−	20.2	1.3	23.6	−	12.0	−	Zn_1.5_Co(CN)_6.0_·2.37H_2_O
DMC-DEP	19.7	9.0	3.9	28.4	3.4	12.2	13.2	1.4	13.8	Zn_1.97_Co(CN)_5.71_·0.82DEP·0.02P123·0.51H_2_O
DMC-P(OEt)_3_	23.0	11.2	3.5	29.0	3.2	13.3	10.2	0.5	13.7	Zn_1.85_Co(CN)_4.98_·0.60P(OEt)_3_·0.01P123·0.14H_2_O
DMC-TEP	16.9	8.3	6.1	30.2	3.9	11.1	21.7	0.9	14.5	Zn_1.84_Co(CN)_5.63_·1.40TEP·0.02P123·0.37H_2_O
DMC-*t*BuOH	23.9	10.7	−	29.3	3.3	16.3	7.2	1.9	24.1	Zn_2.0_Co(CN)_6.4_·0.53*t*BuOH· 0.02P123·0.58H_2_O

**Table 2:** Results for the copolymerization of PO and CO_2_.

Entry	Catalyst	Reaction Condition		Polyol Properties				
		*n*_I_ (mmol)	$P_{{\mathrm{CO}}_{2}}$ (bar)	Conv. (%)	Yield of PPC (%)	$S_{{\mathrm{CO}}_{2}}$^a^ (%)	*M*_n_ (g mol^−1^)	*Ð*
1	DMC-DEP	0.25	30	99.9	61	37	4100	4.36
2	DMC-DEP	2.5	10	90.1	76.5	20	3400	4.33
3	DMC-DEP	2.5	20	99.4	68.1	31	4200	3.81
4	DMC-DEP	2.5	30	99.7	63	33	2000	4.23
5	DMC-DEP	12.5	30	99.6	60	20	1200	2.09
6	DMC-P(OEt)_3_	2.5	30	97.8	56	33	2400	3.47
7	DMC-TEP	2.5	30	97.3	57	34	1900	2.64

^a^ Carbonate content in PPC. Condition: Catalyst amount = 50 mg, PO = 0.3 mol, toluene = 10 mL, *T_P_* = 105 °C, *t* = 3 h.

**Table 3:** Results for the polymerization of PO using various types of macroinitiator.

Block Copolymer	Reaction Condition ^a^					Polyol Properties	
	Initiator			Σ*n*_PO_ (mol)	*t* (min)	*M*_n_ (g mol^−1^)	*Ð*
	Type	*M*_n_ ^b^ (g mol^−1^)	*Ð*				
BCP-1	PCL	2000	1.65	0.45	27	4600	1.26
BCP-2	PCL	1700	1.49	0.35	18.5	3700	1.37
BCP-3	PCL	1700	1.49	0.85	18	6600	1.29
BCP-4	PTMG	1400	1.8	0.1	10	1800	1.23
BCP-5	PTMG	1400	1.8	0.4	12	3600	1.16

^a^ Initiator loading (Σ*n*_I_) = 0.01 mol, Catalyst amount = 50 mg, *T*_P_ = 115 °C. ^b^ Number average molecular weight measured by GPC.

## Data Availability

Data are contained within the article and Supplementary Materials.

## Supplementary Materials

The following supporting information can be downloaded at: https://www.mdpi.com/article/10.3390/polym16060818/s1, Figures S1–S46, Tables S1–S6: FTIR spectra, XRD patterns, SEM images, and TGA curves of DMC catalysts; polymerization rate curves, ^1^H NMR spectra, and GPC curves of polyols. Reference [43] is cited in the Supplementary Materials.
