# Supplementary material for: Synthesis of Polyether, Poly(Ether Carbonate) and Poly(Ether Ester) Polyols Using Double Metal Cyanide Catalysts Bearing Organophosphorus Complexing Agents
Source: Polymers (Basel). 2024 Mar 14;16(6):818. doi: 10.3390/polym16060818 (PMC10975066; doi:10.3390/polym16060818)
Supplement: Supplementary file 1 [file polymers-16-00818-s001.zip › polymers-2895965-supplementary.pdf]

Supporting Information

Synthesis of polyether, poly(ether carbonate) and poly(ether ester) polyols using double metal cyanide catalysts bearing organophosphorus complexing agents

Eun-Gyeong Lee, Chinh Hoang Tran, Ju-Yeong Heo, So-Young Kim, Ha-Kyung Choi, Byeong-Ryeol Moon, and Il Kim *

School of Chemical Engineering, Pusan National University, Busandaehag-ro 63-2, Geumjeong-gu, Busan 46241, Republic of Korea.

***Corresponding Author**.

E-mail address: ilkim@pusan.ac.kr; Tel.: +82-51-510-2466.

Table of Contents

1. Supplementary figures 6

1.1 Characterization of DMC catalysts 6

Fig. S1 FTIR spectra of the DMC-DEP prepared using various temperature and DMC-H_3PO_3 6

Fig. S2 FTIR spectra of the DMC-$P(OEt)_3$ prepared at various temperatures and without co-CA. 6

Fig. S3 FTIR spectra of the DMC-TEP prepared using various temperature. 7

Fig. S4 FTIR spectra of the DMC-TEP prepared using various amounts of CA. 7

Fig. S5 FTIR spectra of the DMC-*t*BuOH prepared using various amounts of CA. 8

Fig. S6 Expanded Zn 2p3 XPS spectra: (a) DMC-pure, (b) DMC-DMMP, (c), (d) DMC-DEP prepared at 30 and 90 °C, respectively, and (e), (f) DMC-$P(OEt)_3$ prepared at 30 and 70 °C, respectively 8

Fig. S7 Expanded Co 2p3 XPS spectra: (a) DMC-pure, (b) DMC-DMMP, (c), (d) DMC-DEP prepared at 30 and 90 °C, respectively, and (e), (f) DMC-$P(OEt)_3$ prepared at 30 and 70 °C, respectively 9

Fig. S8 XRD patterns of the prepared DMC catalysts. 9

Fig. S9 XRD patterns of the DMC-DEP and $P(OEt)_3$ prepared at various temperature. (◯) denote the monoclinic (*P*11*m*) phases. 10

Fig. S10 XRD patterns of the DMC-TEP and DMC-DEP prepared using various amounts of CAs. (□), and (◯) denote the cubic (*Fm*-3*m*), and monoclinic (*P*11*m*) phases, respectively. 10

Fig. S11 SEM images of the DMC-pure and the optimized DMC-DEP catalysts. 11

Fig. S12 TGA curve of the prepared DMC catalysts 11

1.2 Catalytic reaction 12

Fig. S13 Reaction rate curves of the ROP of PO obtained by DMC-$P(OEt)_3$ prepared using various amount of CA and catalyst preparation temperature. Reaction condition: Catalyst loading (n_{Zn}) = 0.3 mmol, PO = 3.5 mol, PPG-600 = 50 mmol, T_P = 115 °C. 12

Fig. S14 Reaction rate curves of the ROP of PO obtained by DMC-DEP prepared using various amount of CA and catalyst preparation temperature. Reaction condition: Catalyst loading (n_{Zn}) = 0.3 mmol, PO = 3.5 mol, PPG-600 = 50 mmol, T_P = 115 °C. 12

Fig. S15 Reaction rate curves of the ROP of PO obtained by DMC-TEP prepared using various amount of CA and catalyst preparation temperature. Reaction condition: Catalyst loading (n_{Zn}) = 0.3 mmol, PO = 3.5 mol, PPG-600 = 50 mmol, T_P = 115 °C. 13

Fig. S16 Reaction rate curves of the ROP of PO obtained by DMC-TEP prepared using various co-CAs. Reaction condition: Catalyst loading (n_{Zn}) = 0.3 mmol, PO = 3.5 mol, PPG-400 = 50 mmol, T_P = 115 °C. 13

Fig. S17 Reaction rate curves of the ROP of PO obtained by DMC-*t*BuOH prepared using various amount of CA. Reaction condition: Catalyst loading (n_{Zn}) = 0.3 mmol, PO = 3.5 mol, PPG-400 = 50 mmol, T_P = 115 °C. 14

Fig. S18 ^{1}H NMR spectrum (400 MHz, $CDCl_3$) of the PPG produced by DMC-DMP. Polymerization condition: Catalyst amount = 100 mg, PO = 200 mol, PPG-600 = 50 mmol, T_P = 115 °C. 14

Fig. S19 ^{1}H NMR spectrum (400 MHz, $CDCl_3$) of the PPG produced by DMC-DEP. Polymerization Reaction condition: Catalyst amount = 100 mg, PO = 200 mol, PPG-600 = 50 mmol, T_P = 115 °C. 15

Fig. S20 ^{1}H NMR spectrum (400 MHz, $CDCl_3$) of the PPG produced by DMC-D*t*BuP. Polymerization Reaction condition: Catalyst amount = 100 mg, PO = 200 mol, PPG-600 = 50 mmol, T_P = 115 °C. 16

Fig. S21 ^{1}H NMR spectrum (400 MHz, $CDCl_3$) of the PPG produced by DMC-$P(OMe)_3$. Polymerization Reaction condition: Catalyst amount = 100 mg, PO = 200 mol, PPG-600 = 50 mmol, T_P = 115 °C. 17

Fig. S22 ^{1}H NMR spectrum (400 MHz, $CDCl_3$) of the PPG produced by DMC-$P(OEt)_3$. Polymerization Reaction condition: Catalyst amount = 100 mg, PO = 200 mol, PPG-600 = 50 mmol, T_P = 115 °C. 18

Fig. S23 ^{1}H NMR spectrum (400 MHz, $CDCl_3$) of the PPG produced by DMC-TEP. Polymerization Reaction condition: Catalyst amount = 100 mg, PO = 200 mol, PPG-600 = 50 mmol, T_P = 115 °C. 19

Fig. S24 ^{1}H NMR spectrum (400 MHz, $CDCl_3$) of the PPG produced by DMC-TIP. Polymerization Reaction condition: Catalyst amount = 100 mg, PO = 200 mol, PPG-600 = 50 mmol, T_P = 115 °C. 20

Fig. S25 ^{1}H NMR spectrum (400 MHz, $CDCl_3$) of the crude reaction mixture of the ROP of PO and CO_2 obtained by DMC-DEP. Polymerization Reaction condition: Catalyst amount = 50 mg , PO = 0.34 mol, PPG-600 = 2.5 mmol, toluene = 10 mL, P_{CO2} = 5 bar, T_P = 105 °C, t_P = 3 h. 21

Fig. S26 ^{1}H NMR spectrum (400 MHz, $CDCl_3$) of the crude reaction mixture of the ROP of PO and CO_2 obtained by DMC-DEP. Polymerization Reaction condition: Catalyst amount = 50 mg , PO = 0.34 mol, PPG-600 = 2.5 mmol, toluene = 10 mL, P_{CO2} = 10 bar, T_P = 105 °C, t_P = 3 h. 22

Fig. S27 ^{1}H NMR spectrum (400 MHz, $CDCl_3$) of the polycarbonate polyol obtained by DMC- DEP. Polymerization Reaction condition: Catalyst amount = 50 mg , PO = 0.34 mol, PPG-600 = 2.5 mmol, toluene = 10 mL, P_{CO2} = 10 bar, T_P = 105 °C, t_P = 3 h. 23

Fig. S28 ^{1}H NMR spectrum (400 MHz, $CDCl_3$) of the crude reaction mixture of the ROP of PO and CO_2 obtained by DMC-DEP. Polymerization Reaction condition: Catalyst amount = 50 mg , PO = 0.34 mol, PPG-600 = 2.5 mmol, toluene = 10 mL, P_{CO2} = 20 bar, T_P = 105 °C, t_P = 3 h. 24

Fig. S29 ^{1}H NMR spectrum (400 MHz, $CDCl_3$) of the polycarbonate polyol obtained by DMC- DEP. Polymerization Reaction condition: Catalyst amount = 50 mg , PO = 0.34 mol, PPG-600 = 2.5 mmol, toluene = 10 mL, P_{CO2} = 20 bar, T_P = 105 °C, t_P = 3 h...................... 25

Fig. S30 ^{1}H NMR spectrum (400 MHz, $CDCl_3$) of the crude reaction mixture of the ROP of PO and CO_2 obtained by DMC-DEP. Polymerization Reaction condition: Catalyst amount = 50 mg , PO = 0.34 mol, PPG-600 = 0.25 mmol, toluene = 10 mL, P_{CO2} = 30 bar, T_P = 105 °C, t_P = 3 h. 27

Fig. S31 ^{1}H NMR spectrum (400 MHz, $CDCl_3$) of the polycarbonate polyol obtained by DMC- DEP. Polymerization Reaction condition: Catalyst amount = 50 mg , PO = 0.34 mol, PPG-600 = 0.25 mmol, toluene = 10 mL, P_{CO2} = 30 bar, T_P = 105 °C, t_P = 3 h.................... 27

Fig. S32 ^{1}H NMR spectrum (400 MHz, $CDCl_3$) of the crude reaction mixture of the ROP of PO and CO_2 obtained by DMC-DEP. Polymerization Reaction condition: Catalyst amount = 50 mg , PO = 0.34 mol, PPG-600 = 2.5 mmol, toluene = 10 mL, P_{CO2} = 30 bar, T_P = 105 °C, t_P = 3 h. 28

Fig. S33 ^{1}H NMR spectrum (400 MHz, $CDCl_3$) of the polycarbonate polyol obtained by DMC- DEP. Polymerization Reaction condition: Catalyst amount = 50 mg , PO = 0.34 mol, PPG-600 = 2.5 mmol, toluene = 10 mL, P_{CO2} = 30 bar, T_P = 105 °C, t_P = 3 h...................... 29

Fig. S34 ^{1}H NMR spectrum (400 MHz, $CDCl_3$) of the crude reaction mixture of the ROP of PO and CO_2 obtained by DMC-DEP. Polymerization Reaction condition: Catalyst amount = 50 mg , PO = 0.34 mol, PPG-600 = 12.5 mmol, toluene = 10 mL, P_{CO2} = 30 bar, T_P = 105 °C, t_P = 3 h. 30

Fig. S35 ^{1}H NMR spectrum (400 MHz, $CDCl_3$) of the polycarbonate polyol obtained by DMC- DEP. Polymerization Reaction condition: : Catalyst amount = 50 mg , PO = 0.34 mol, PPG-600 = 12.5 mmol, toluene = 10 mL, P_{CO2} = 30 bar, T_P = 105 °C, t_P = 3 h. 31

Fig. S36 ^{1}H NMR spectrum (400 MHz, $CDCl_3$) of the crude reaction mixture of the ROP of PO and CO_2 obtained by DMC-$P(OEt)_3$. Polymerization Reaction condition: Catalyst amount = 50 mg , PO = 0.34 mol, PPG-600 = 2.5 mmol, toluene = 10 mL, P_{CO2} = 30 bar, T_P = 105 °C, t_P = 3 h.. 32

Fig. S37 ^{1}H NMR spectrum (400 MHz, $CDCl_3$) of the polycarbonate polyol obtained by DMC- $P(OEt)_3$. Polymerization Reaction condition: : Catalyst amount = 50 mg , PO = 0.34 mol, PPG-600 = 2.5 mmol, toluene = 10 mL, P_{CO2} = 30 bar, T_P = 105 °C, t_P = 3 h. 33

Fig. S38 ^{1}H NMR spectrum (400 MHz, $CDCl_3$) of the crude reaction mixture of the ROP of PO and CO_2 obtained by DMC-TEP. Polymerization Reaction condition: : Catalyst amount = 50 mg , PO = 0.34 mol, PPG-600 = 2.5 mmol, toluene = 10 mL, P_{CO2} = 30 bar, T_P = 105 °C, t_P = 3 h 34

Fig. S39 ^{1}H NMR spectrum (400 MHz, $CDCl_3$) of the polycarbonate polyol obtained by DMC- TEP. Polymerization Reaction condition: : Catalyst amount = 50 mg , PO = 0.34 mol, PPG-600 = 2.5 mmol, toluene = 10 mL, P_{CO2} = 30 bar, T_P = 105 °C, t_P = 3 h...................... 35

Fig. S40 ^{1}H NMR spectrum (400 MHz, $CDCl_3$) of the crude reaction mixture of the ROP of PO and CO_2 obtained by DMC-TEP without co-CA. Polymerization Reaction condition: :

Catalyst amount = 50 mg , PO = 0.34 mol, PPG-600 = 2.5 mmol, toluene = 10 mL, P_{CO2} = 30 bar, T_P = 105 °C, t_P = 3 h 36

Fig. S41 ^{1}H NMR spectrum (400 MHz, $CDCl_3$) of the polycarbonate polyol obtained by DMC- TEP without co-CA. Polymerization Reaction condition: Catalyst amount = 50 mg , PO = 0.34 mol, PPG-600 = 2.5 mmol, toluene = 10 mL, P_{CO2} = 30 bar, T_P = 105 °C, t_P = 3 h. 37

Fig. S42 ^{1}H NMR spectra (400 MHz, $CDCl_3$) of the crude reaction mixture of CL: polymerization using EG initiator and DMC-DEP catalyst. Reaction Conditions: catalyst amount = 10 mg ([Zn]0 = 30 mM), [CL]0 = 9 M, [CL]0/[EG]0 = 10, *Tp* = 160 °C........ 38

Fig. S43 2D DOSY NMR Spectra (600 MHz, $CDCl_3$) of (a) PCL 2000 and (b) PPG. Reaction condition: Catalyst amount = 50 mg, T_P = 115 °C. 38

Fig. S44 ^{1} H-NMR spectra of PPG, PTMG and PPG-PTMG block copolymer (BCP-5)..... 39

Fig. S45 2D DOSY NMR Spectra (600 MHz, $CDCl_3$) of (a) PPG and PTMG mixture and (b) BCP-5. Reaction condition: Catalyst amount = 50 mg, T_P = 115 °C........ 39

Fig. S46 GPC curves of the block copolymer obtained by batch ROP initiated by (a) PCL and (b) PTMG........ 40

2. Supplementary tables 41

Table S1. DMC catalysts prepared using $ZnCl_2$(15 mmol), $K_3Co(CN)_6$ (1.5 mmol), and various type of OPC CAs. 41

Table S2 Properties of triblock copolymers as co-complexing agent........ 42

Table S3 Summary of the FTIR results of DMC catalysts prepared by different OPC CAs 43

Table S4 Summary of the FTIR results of optimized DMC catalysts prepared by different temperature 44

Table S5 Summary of the XPS results of DMC catalysts prepared by different CAs.......... 45

Table S6 Results for the semi-batch ROP of PO using various DMC catalysts........ 46

3. References........ 48

1. Supplementary figures

1.1 Characterization of DMC catalysts

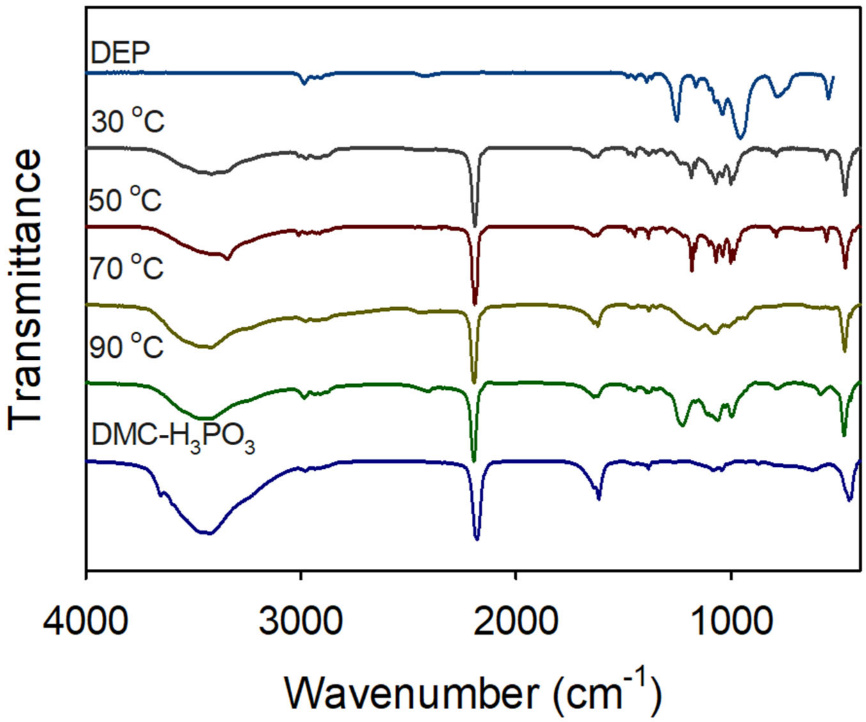

Fig. S1 FTIR spectra of the DMC-DEP prepared using various temperature and DMC-H_3PO_3.

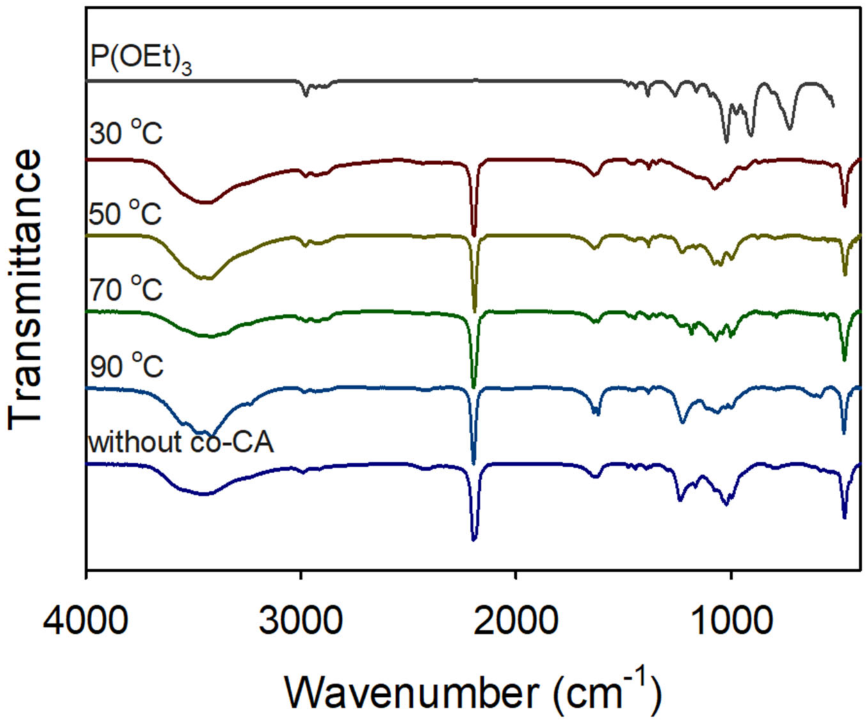

Fig. S2 FTIR spectra of the DMC-P(OEt)$_3$ prepared at various temperatures and without co-CA.

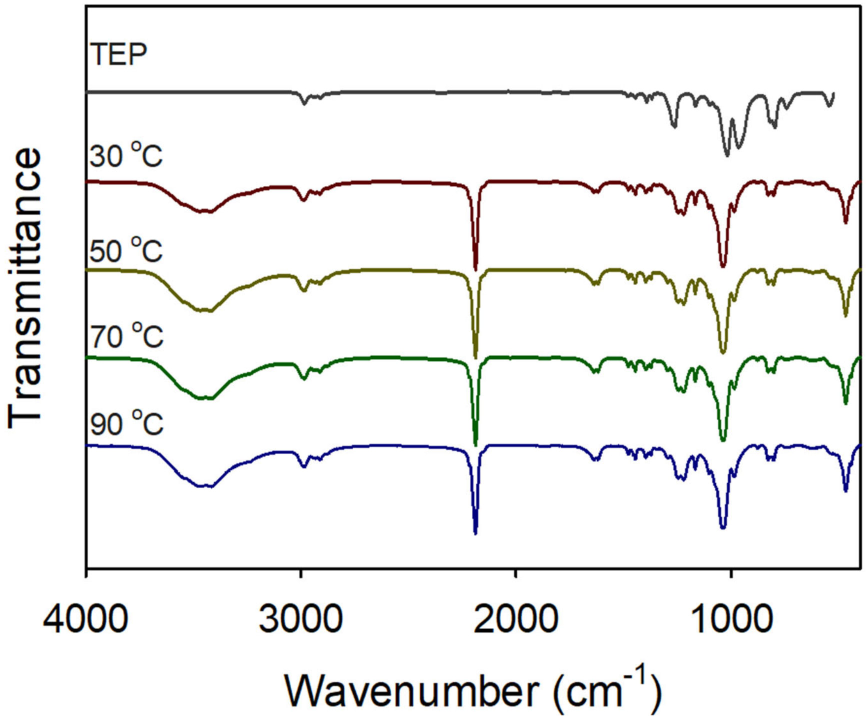

Fig. S3 FTIR spectra of the DMC-TEP prepared using various temperature.

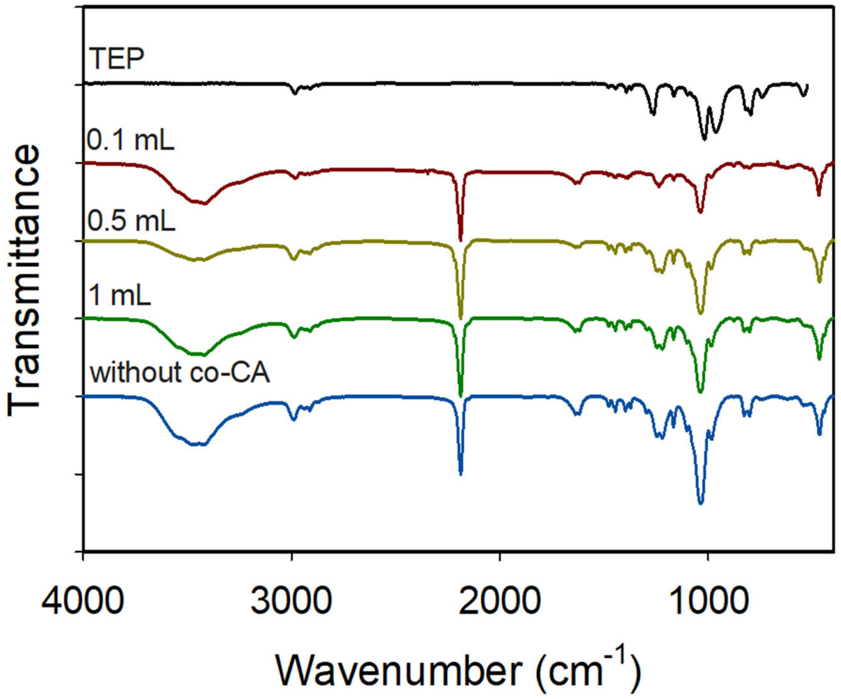

Fig. S4 FTIR spectra of the DMC-TEP prepared using various amounts of CA.

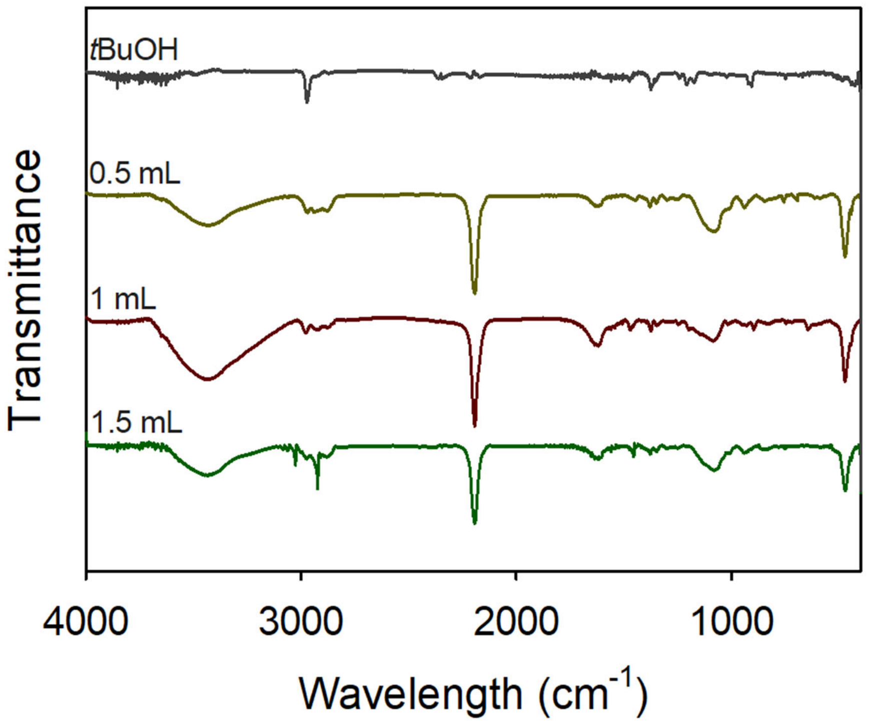

Fig. S5 FTIR spectra of the DMC-*t*BuOH prepared using various amounts of CA.

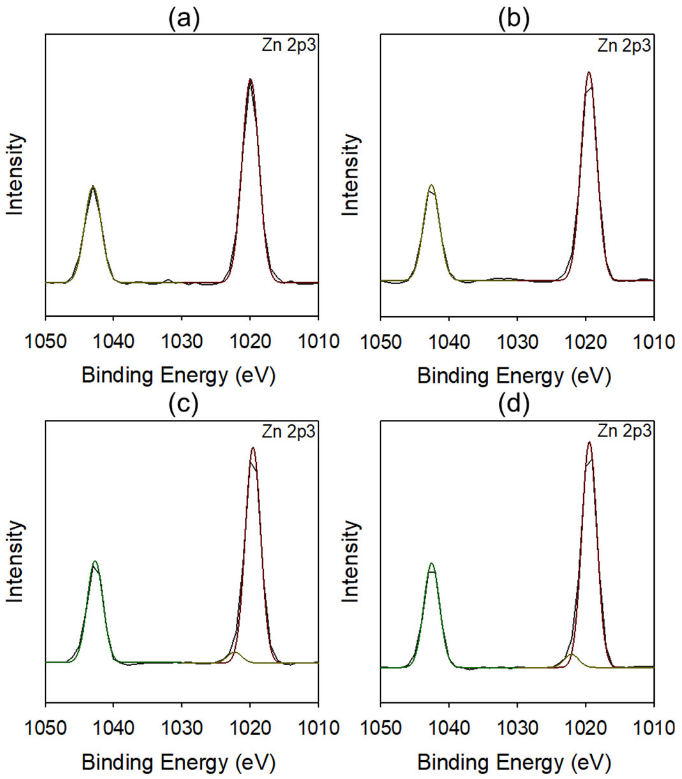

Fig. S6 Expanded Zn 2p3 XPS spectra: (a) DMC-pure, (b) DMC-DMMP, (c) DMC-DEP, and (d) DMC-$P(OEt)_3$.

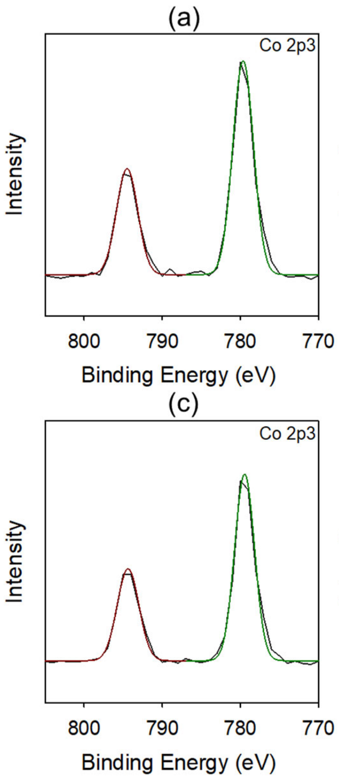

Fig. S7 Expanded Co 2p3 XPS spectra: (a) DMC-pure, (b) DMC-DMMP, (c) DMC-DEP, and (d) DMC-P(OEt)3.

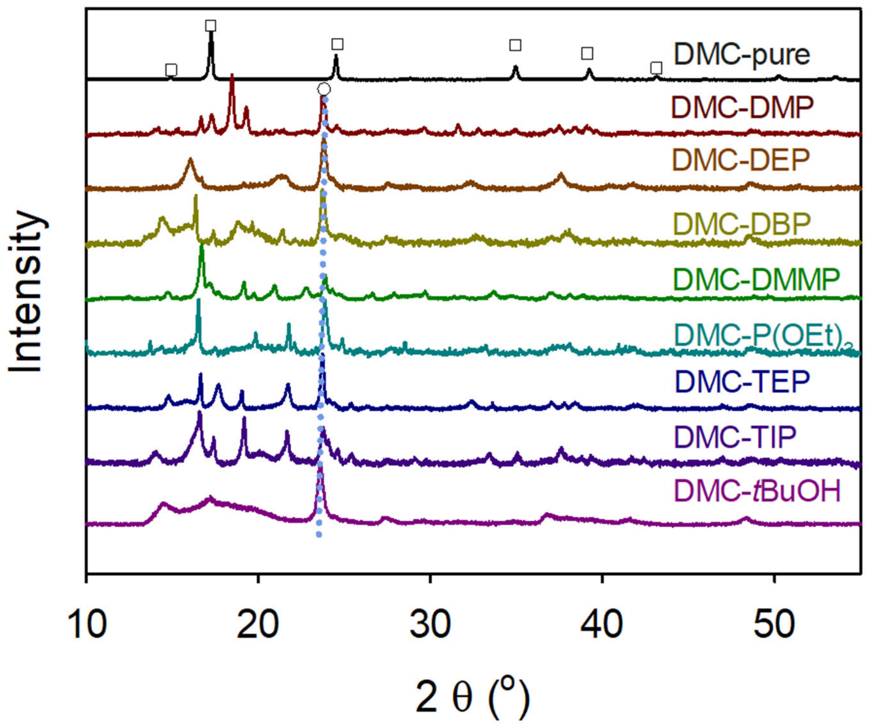

Fig. S8 XRD patterns of the prepared DMC catalysts.

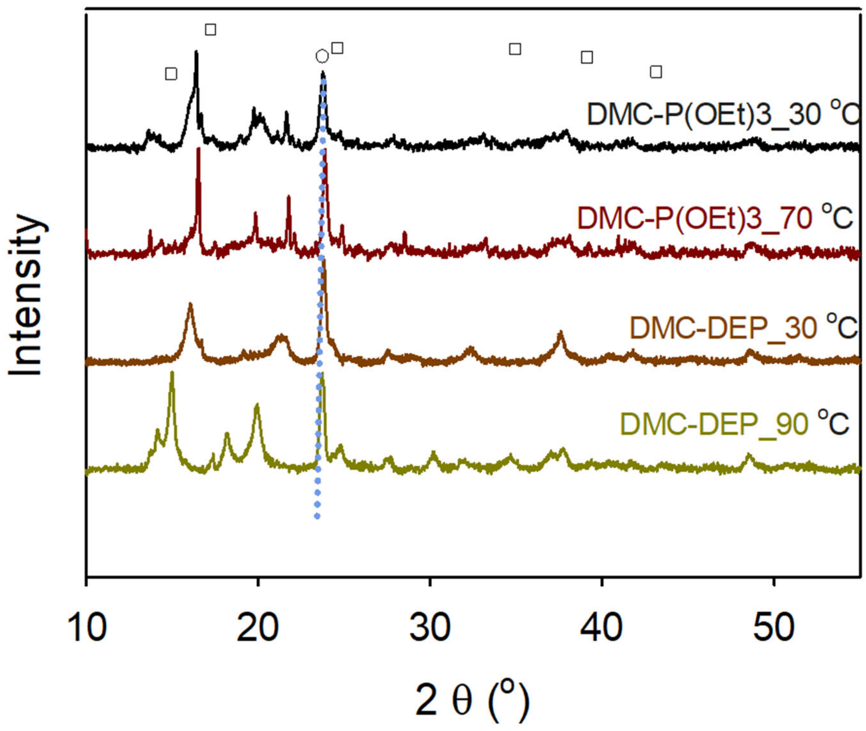

Fig. S9 XRD patterns of the DMC-DEP and P(OEt)$_3$ prepared at various temperature. (○) denote the monoclinic (*P*11*m*) phases.

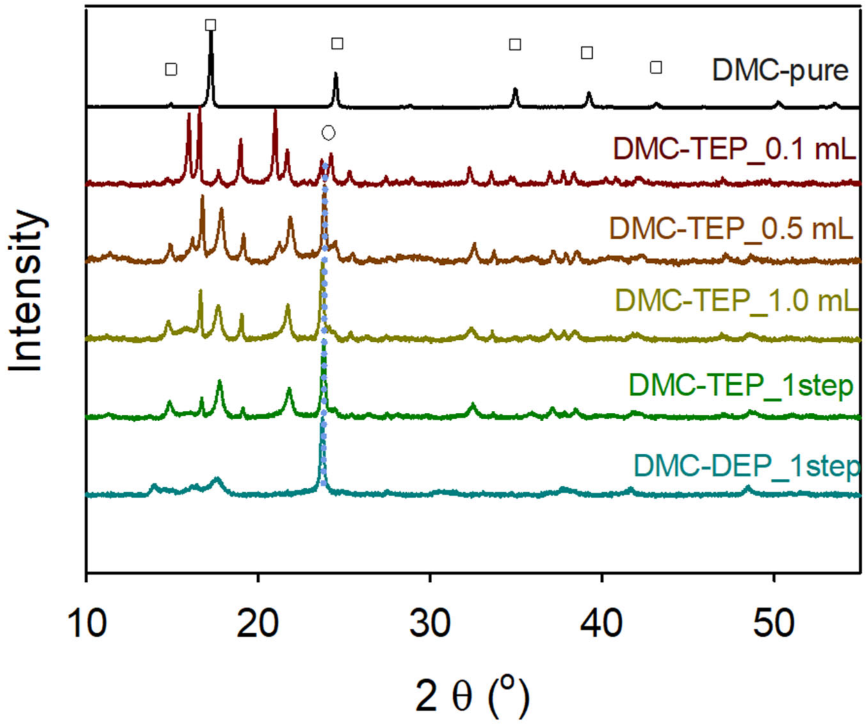

Fig. S10 XRD patterns of the DMC-TEP and DMC-DEP prepared using various amounts of CAs. (□), and (○) denote the cubic (*Fm*-3*m*), and monoclinic (*P*11*m*) phases, respectively.

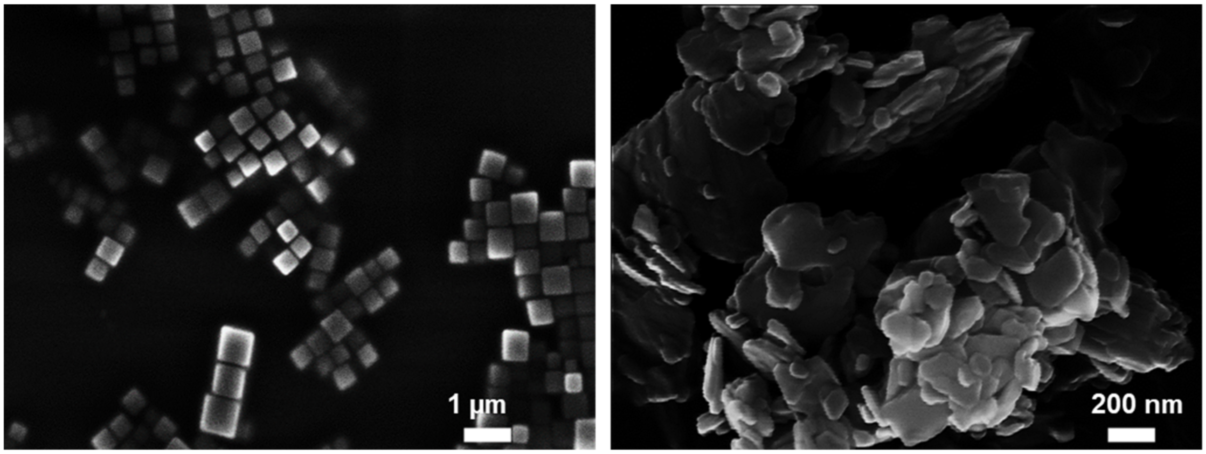

Fig. S11 SEM images of the DMC-pure and the optimized DMC-DEP catalysts.

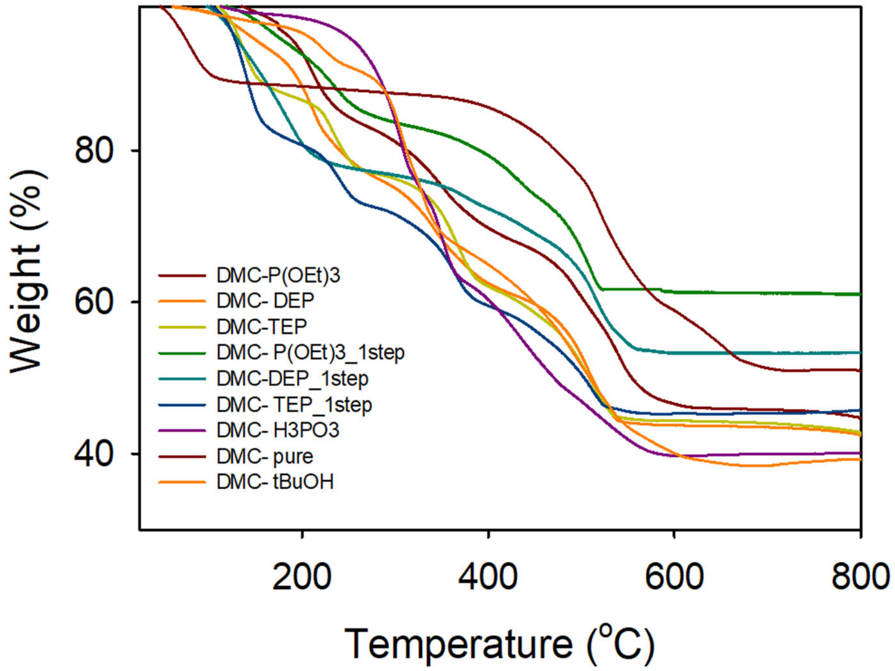

Fig. S12 TGA curve of the prepared DMC catalysts

1.2 Catalytic reaction

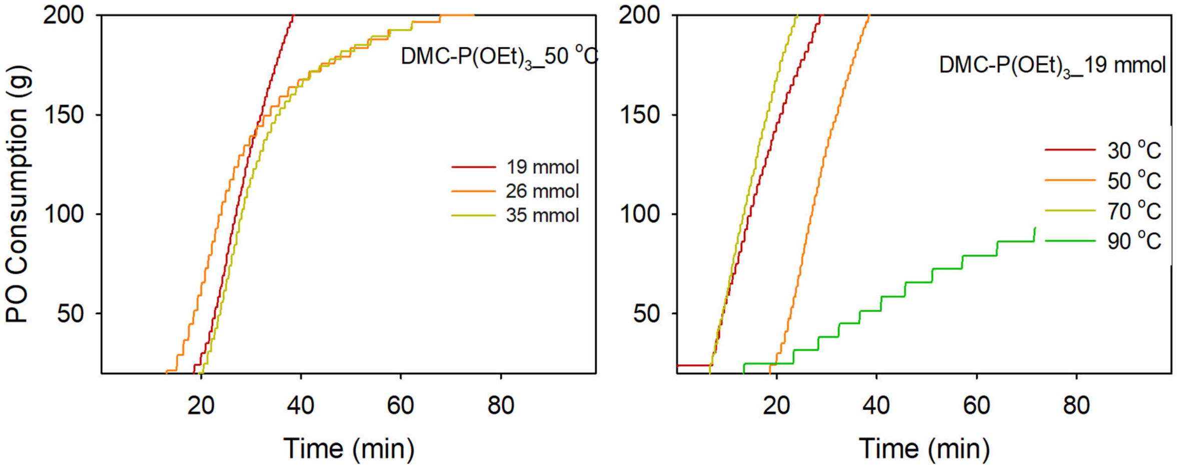

Fig. S13 Reaction rate curves of the ROP of PO obtained by DMC-P(OEt)3 prepared using various amount of CA and catalyst preparation temperature. Reaction condition: Catalyst loading (n_{Zn}) = 0.3 mmol, PO = 3.5 mol, PPG-600 = 50 mmol, T_P = 115 °C.

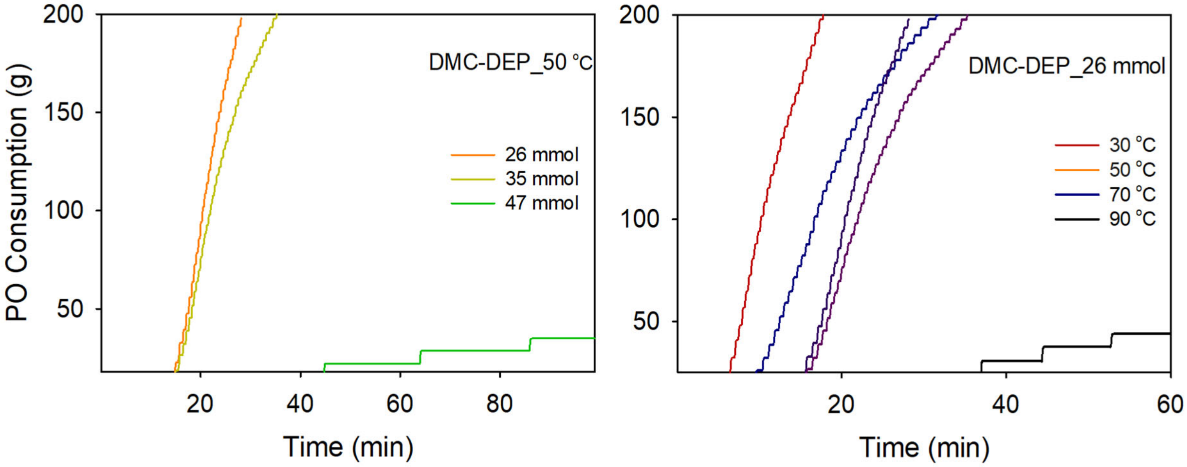

Fig. S14 Reaction rate curves of the ROP of PO obtained by DMC-DEP prepared using various amount of CA and catalyst preparation temperature. Reaction condition: Catalyst loading (n_{Zn}) = 0.3 mmol, PO = 3.5 mol, PPG-600 = 50 mmol, T_P = 115 °C.

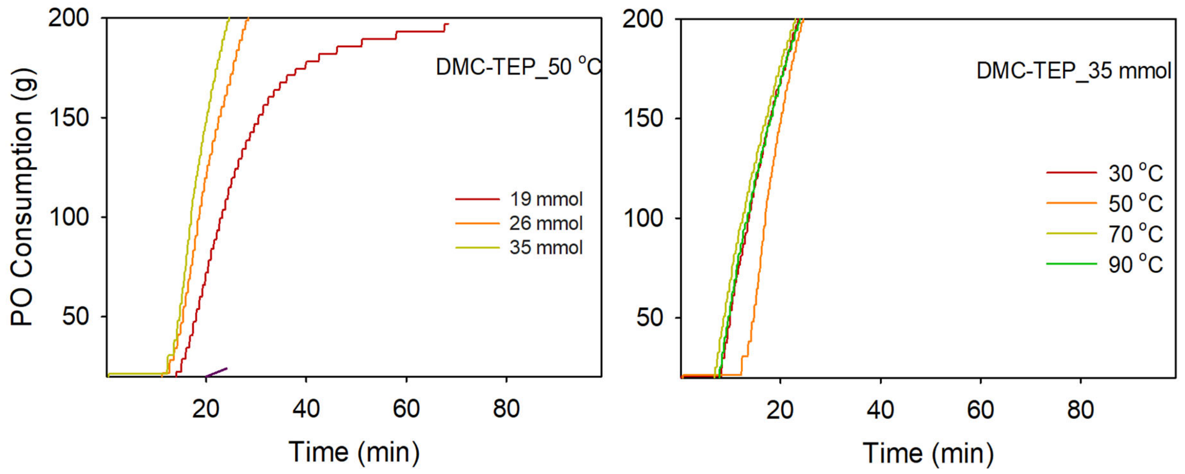

Fig. S15 Reaction rate curves of the ROP of PO obtained by DMC-TEP prepared using various amount of CA and catalyst preparation temperature. Reaction condition: Catalyst loading (n_{Zn}) = 0.3 mmol, PO = 3.5 mol, PPG-600 = 50 mmol, T_P = 115 °C.

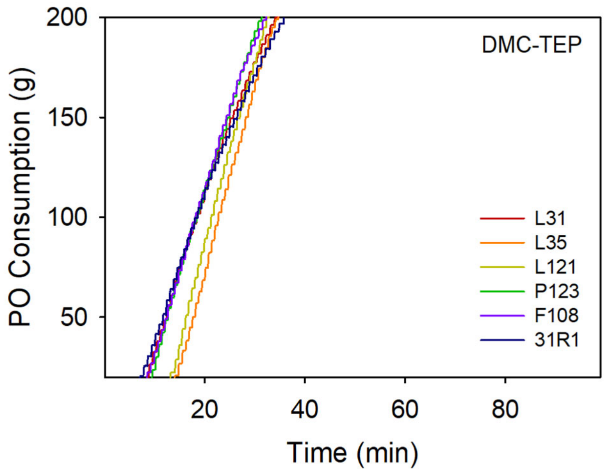

Fig. S16 Reaction rate curves of the ROP of PO obtained by DMC-TEP prepared using various co-CAs. Reaction condition: Catalyst loading (n_{Zn}) = 0.3 mmol, PO = 3.5 mol, PPG-400 = 50 mmol, T_P = 115 °C.

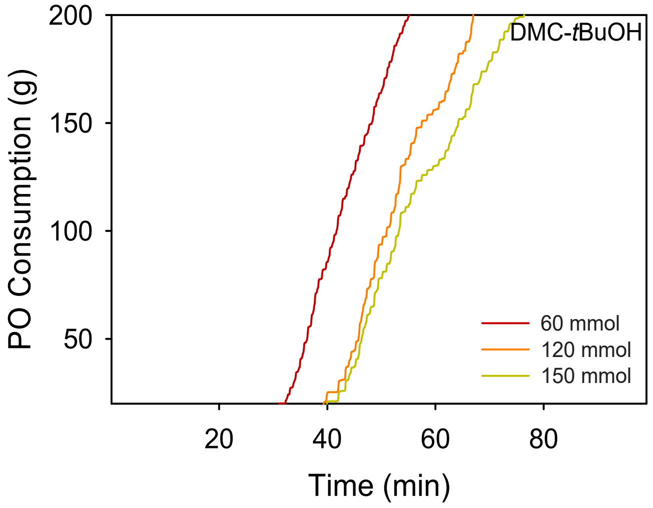

Fig. S17 Reaction rate curves of the ROP of PO obtained by DMC-*t*BuOH prepared using various amount of CA. Reaction condition: Catalyst loading (n_{Zn}) = 0.3 mmol, PO = 3.5 mol, PPG-400 = 50 mmol, T_P = 115 °C.

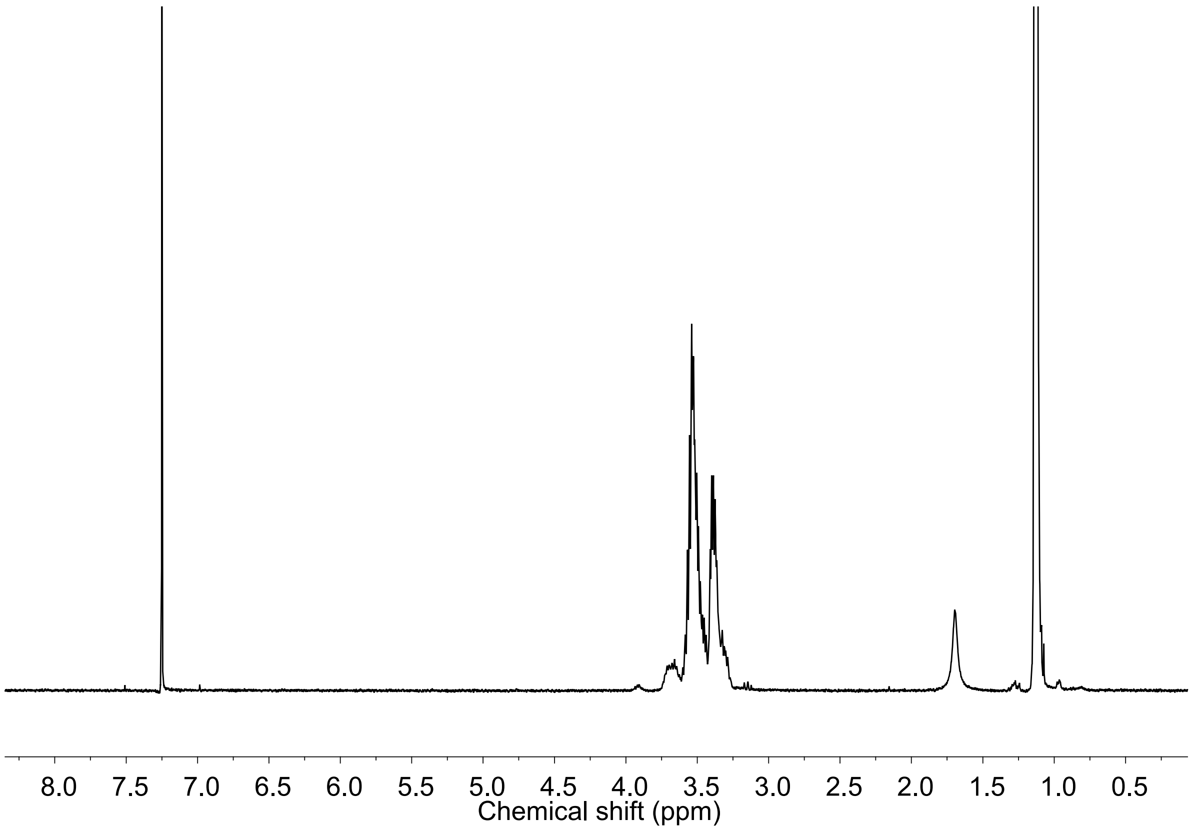

Fig. S18 ^{1}H NMR spectrum (400 MHz, $CDCl_3$) of the PPG produced by DMC-DMP. Polymerization condition: Catalyst amount = 100 mg, PO = 200 mol, PPG-600 = 50 mmol, T_P = 115 °C.

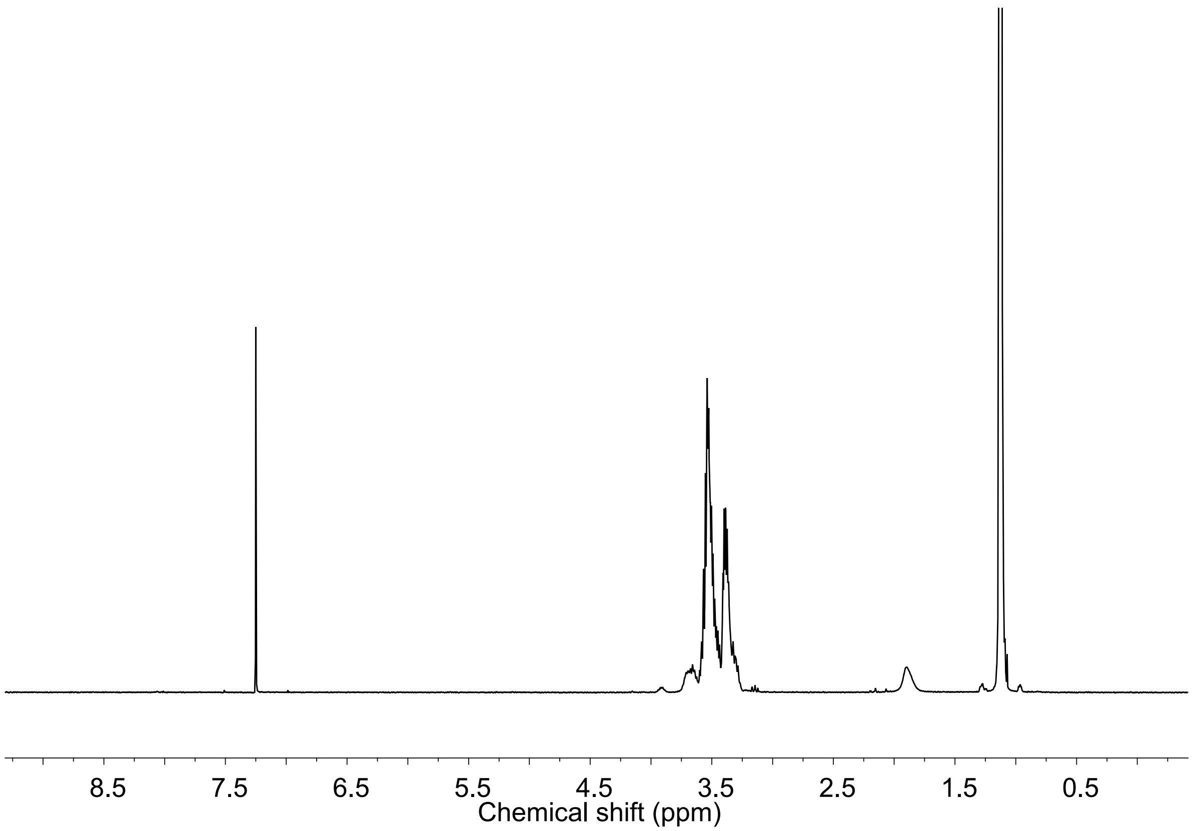

Fig. S19 ^{1}H NMR spectrum (400 MHz, $CDCl_3$) of the PPG produced by DMC-DEP. Polymerization Reaction condition: Catalyst amount = 100 mg, PO = 200 mol, PPG-600 = 50 mmol, T_P = 115 °C.

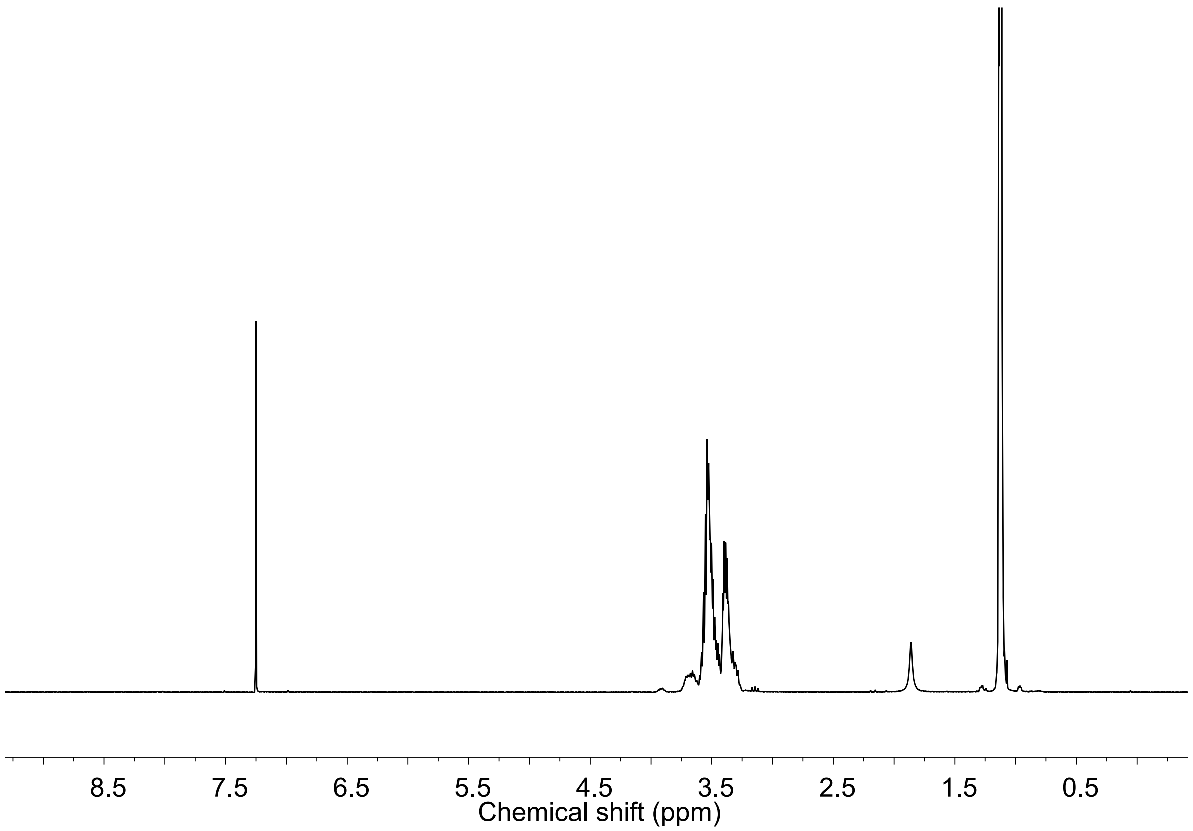

Fig. S20 ^{1}H NMR spectrum (400 MHz, $CDCl_3$) of the PPG produced by DMC-D*t*BuP. Polymerization Reaction condition: Catalyst amount = 100 mg, PO = 200 mol, PPG-600 = 50 mmol, T_P = 115 °C.

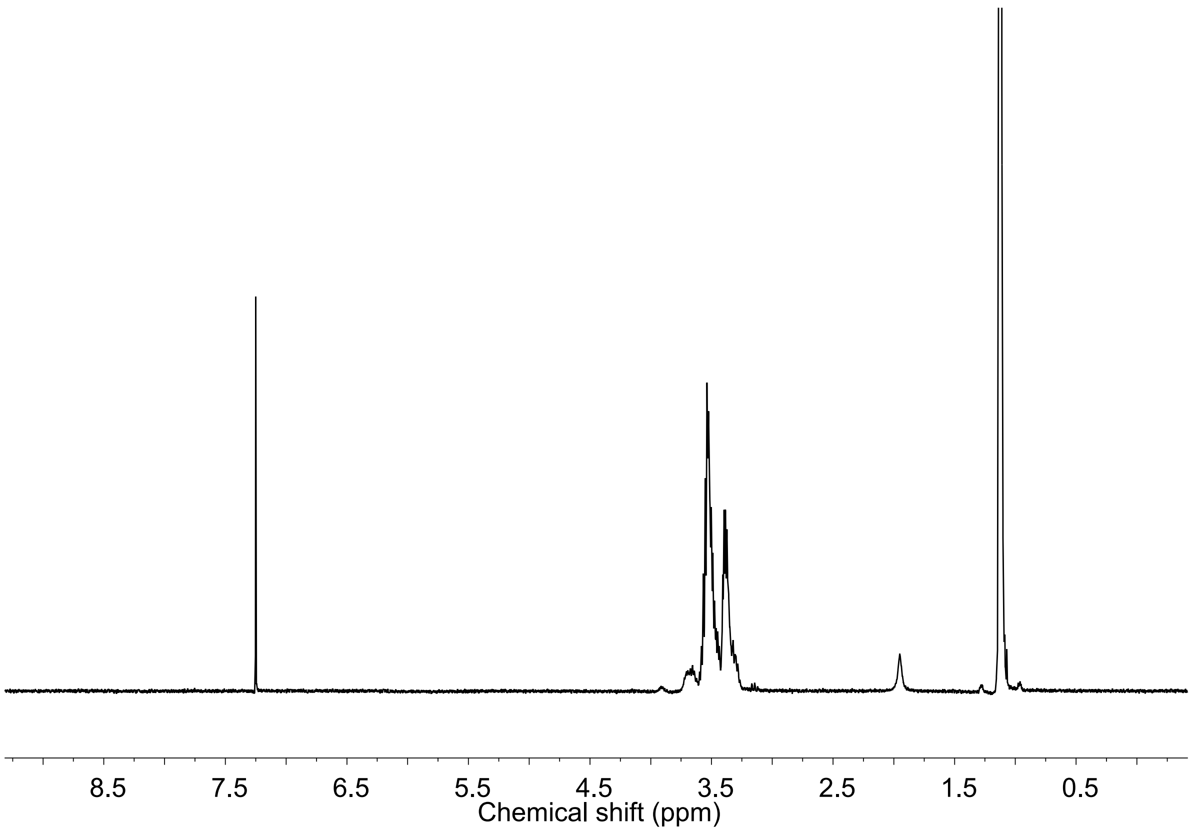

Fig. S21 ^{1}H NMR spectrum (400 MHz, $CDCl_3$) of the PPG produced by DMC-$P(OMe)_3$. Polymerization Reaction condition: Catalyst amount = 100 mg, PO = 200 mol, PPG-600 = 50 mmol, T_P = 115 °C.

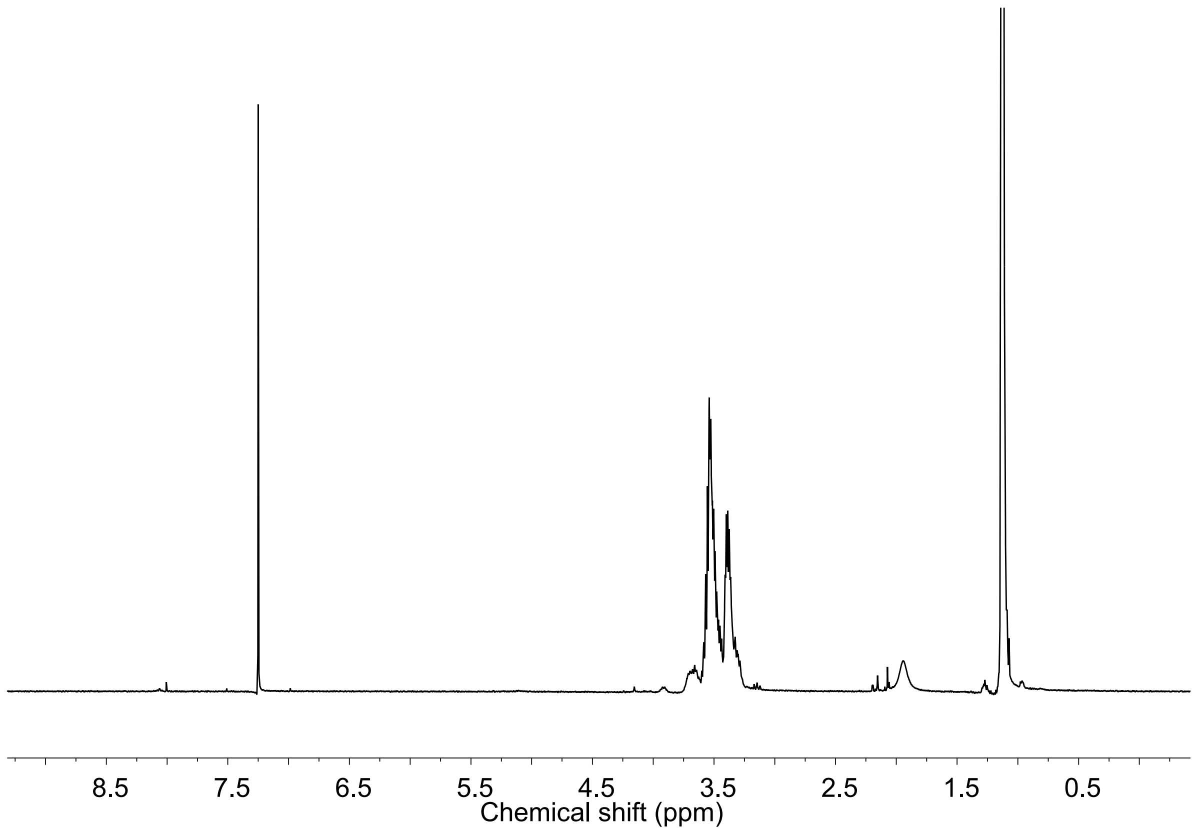

Fig. S22 ^{1}H NMR spectrum (400 MHz, $CDCl_3$) of the PPG produced by DMC-P(OEt)$_3$. Polymerization Reaction condition: Catalyst amount = 100 mg, PO = 200 mol, PPG-600 = 50 mmol, T_P = 115 °C.

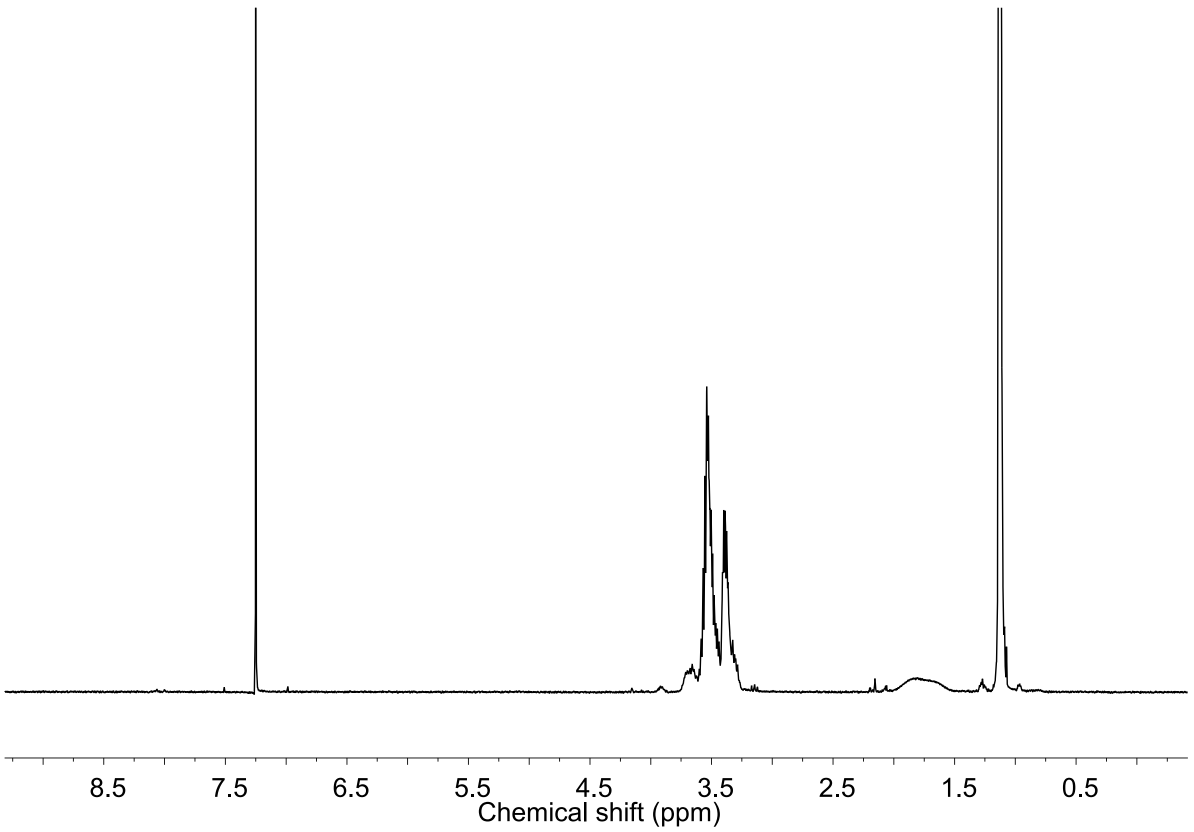

Fig. S23 ^{1}H NMR spectrum (400 MHz, $CDCl_3$) of the PPG produced by DMC-TEP. Polymerization Reaction condition: Catalyst amount = 100 mg, PO = 200 mol, PPG-600 = 50 mmol, T_P = 115 °C.

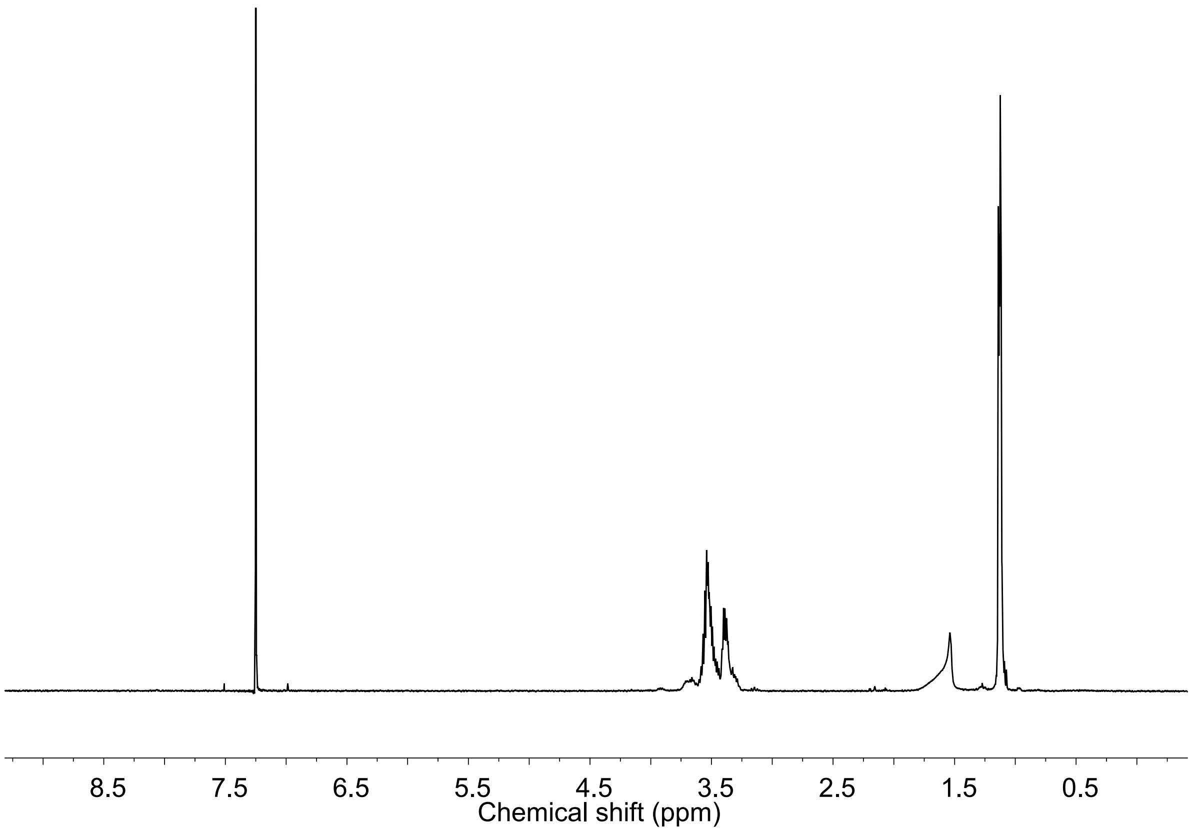

Fig. S24 ^{1}H NMR spectrum (400 MHz, $CDCl_3$) of the PPG produced by DMC-TIP. Polymerization Reaction condition: Catalyst amount = 100 mg, PO = 200 mol, PPG-600 = 50 mmol, T_P = 115 °C.

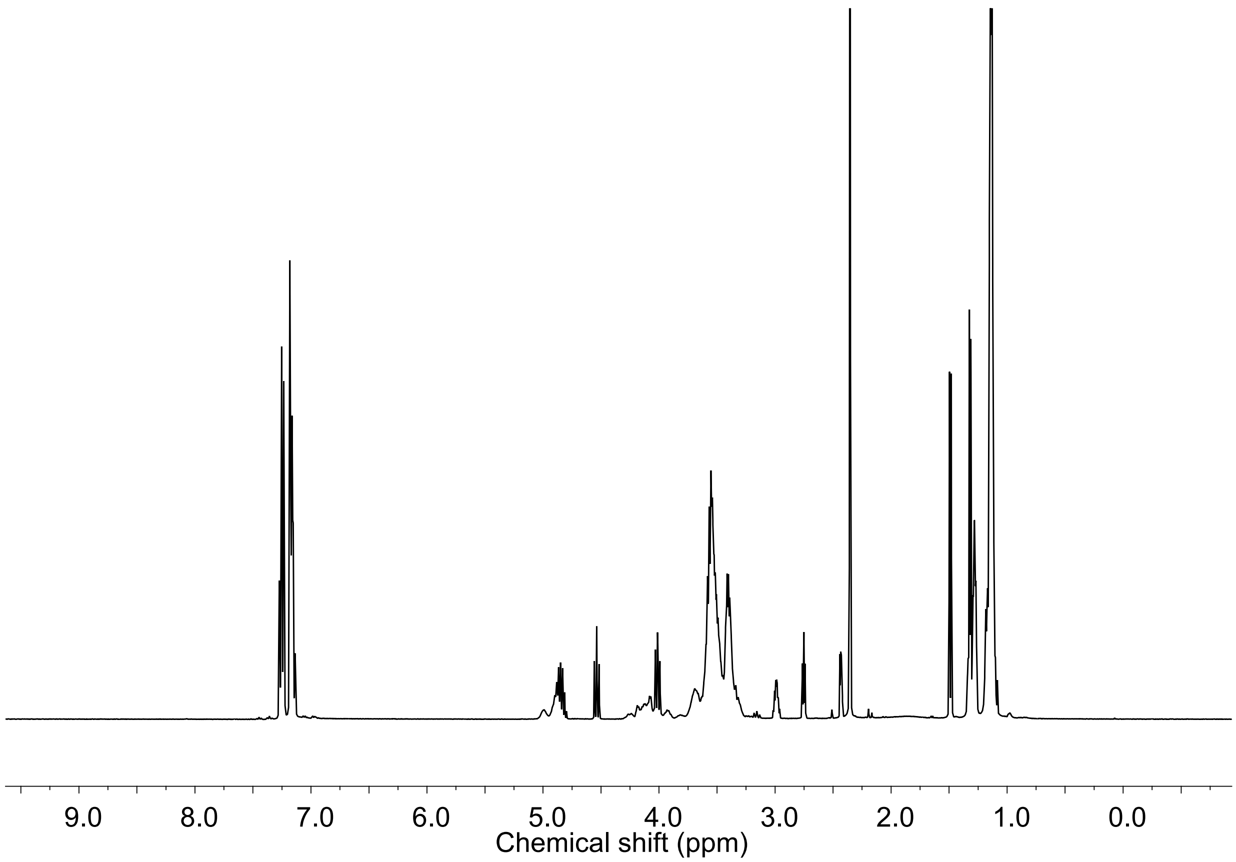

Fig. S25 ^{1}H NMR spectrum (400 MHz, $CDCl_3$) of the crude reaction mixture of the ROP of PO and CO_2 obtained by DMC-DEP. Polymerization Reaction condition: Catalyst amount = 50 mg , PO = 0.34 mol, PPG-600 = 2.5 mmol, toluene = 10 mL, P_{CO_2} = 5 bar, T_P = 105 °C, t_P = 3 h.

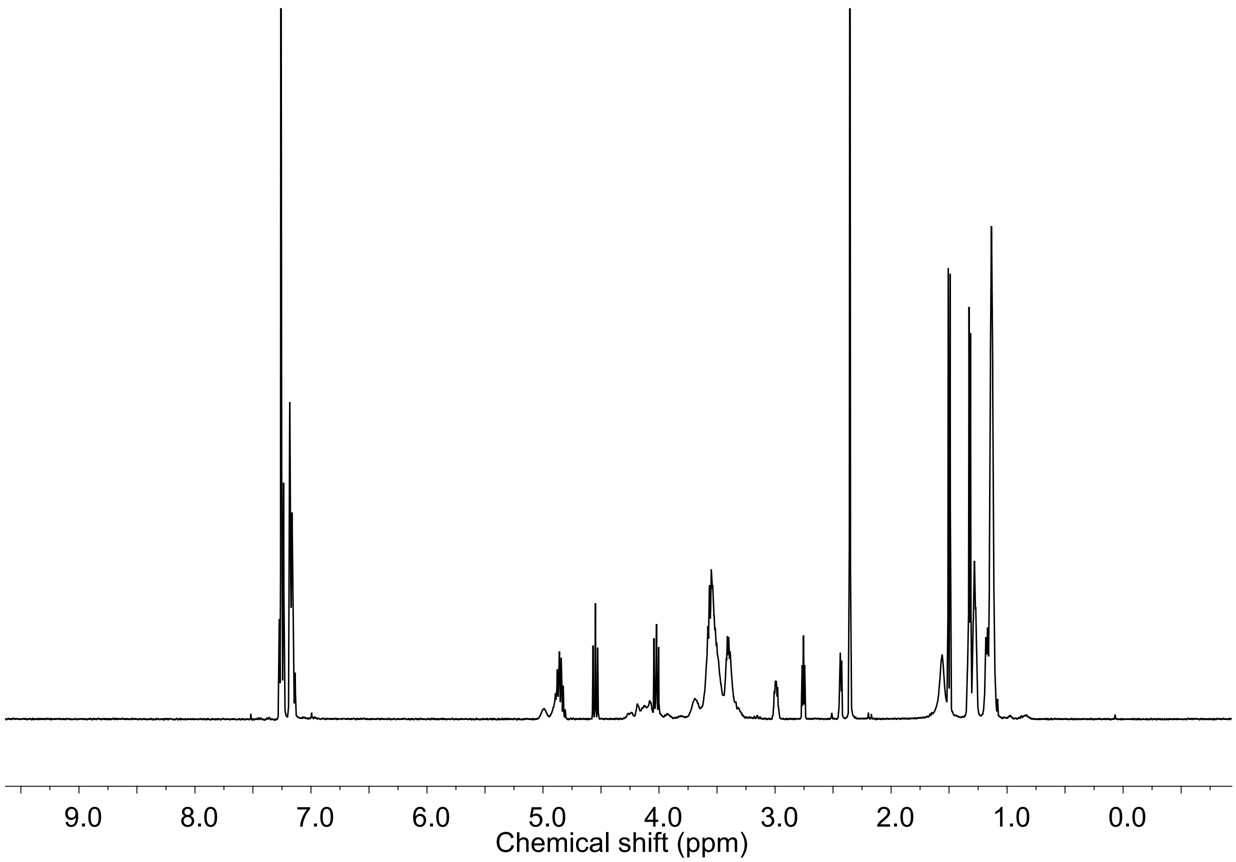

Fig. S26 ^{1}H NMR spectrum (400 MHz, $CDCl_3$) of the crude reaction mixture of the ROP of PO and CO_2 obtained by DMC-DEP. Polymerization Reaction condition: Catalyst amount = 50 mg , PO = 0.34 mol, PPG-600 = 2.5 mmol, toluene = 10 mL, P_{CO_2} = 10 bar, T_P = 105 °C, t_P = 3 h.

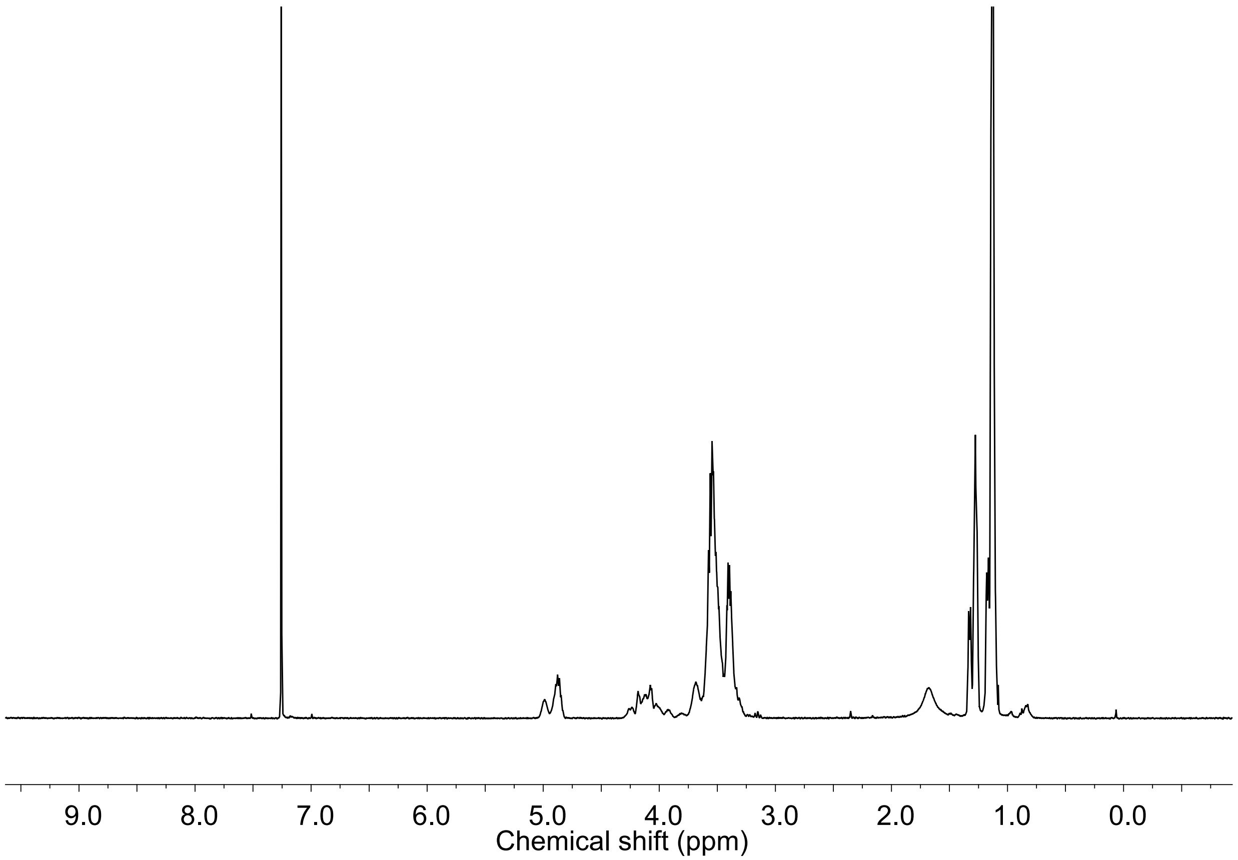

Fig. S27 ^{1}H NMR spectrum (400 MHz, $CDCl_3$) of the polycarbonate polyol obtained by DMC-DEP. Polymerization Reaction condition: Catalyst amount = 50 mg , PO = 0.34 mol, PPG-600 = 2.5 mmol, toluene = 10 mL, P_{CO_2} = 10 bar, T_P = 105 °C, t_P = 3 h.

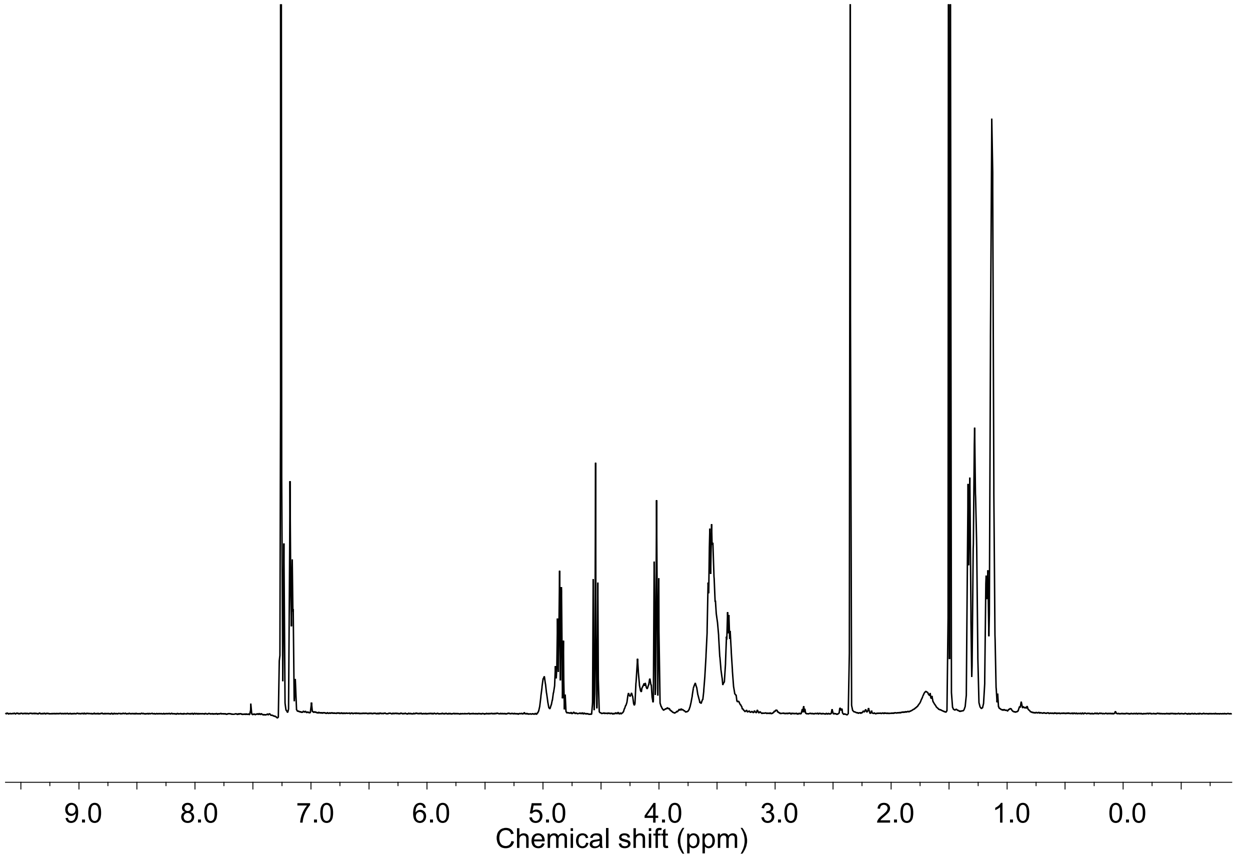

Fig. S28 ^{1}H NMR spectrum (400 MHz, $CDCl_3$) of the crude reaction mixture of the ROP of PO and CO_2 obtained by DMC-DEP. Polymerization Reaction condition: Catalyst amount = 50 mg , PO = 0.34 mol, PPG-600 = 2.5 mmol, toluene = 10 mL, P_{CO_2} = 20 bar, T_P = 105 °C, t_P = 3 h.

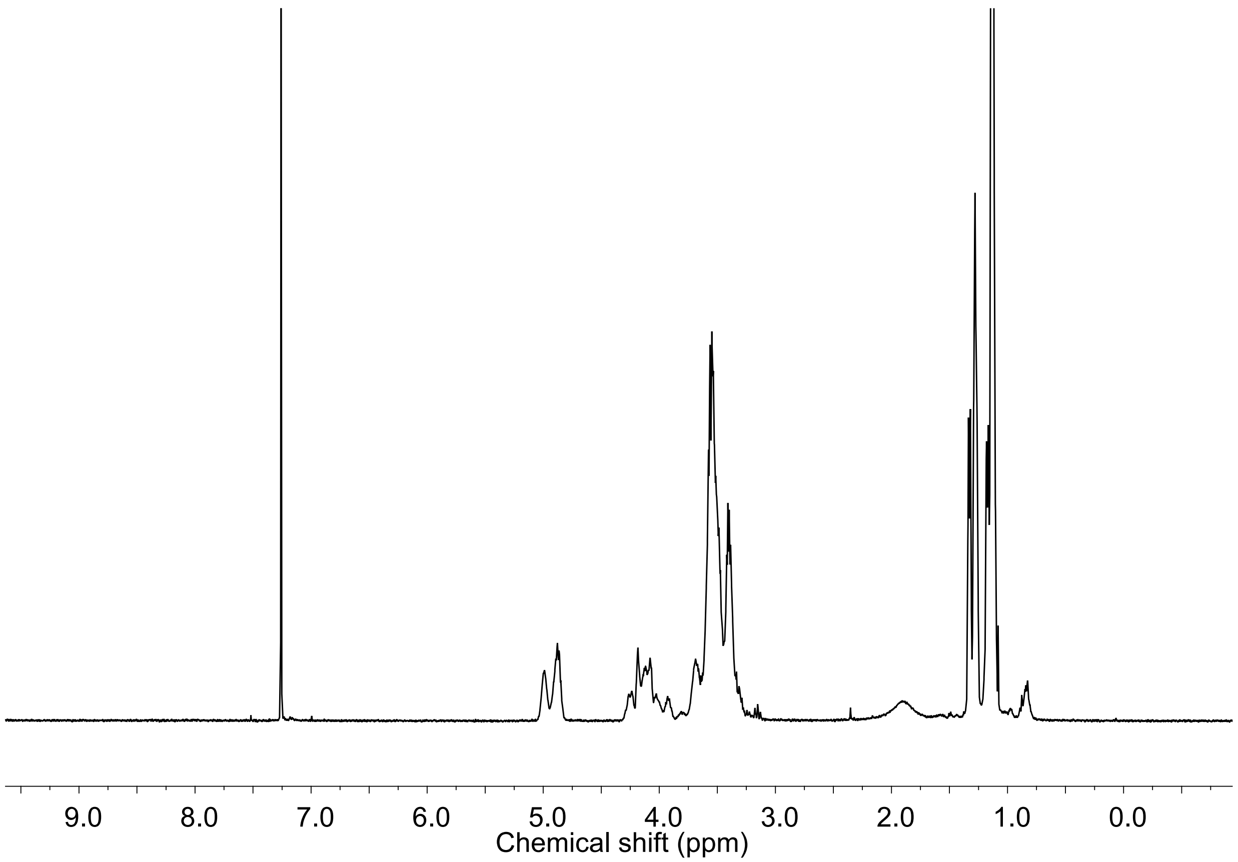

Fig. S29 ^{1}H NMR spectrum (400 MHz, $CDCl_3$) of the polycarbonate polyol obtained by DMC-DEP. Polymerization Reaction condition: Catalyst amount = 50 mg , PO = 0.34 mol, PPG-600 = 2.5 mmol, toluene = 10 mL, P_{CO_2} = 20 bar, T_P = 105 °C, t_P = 3 h.

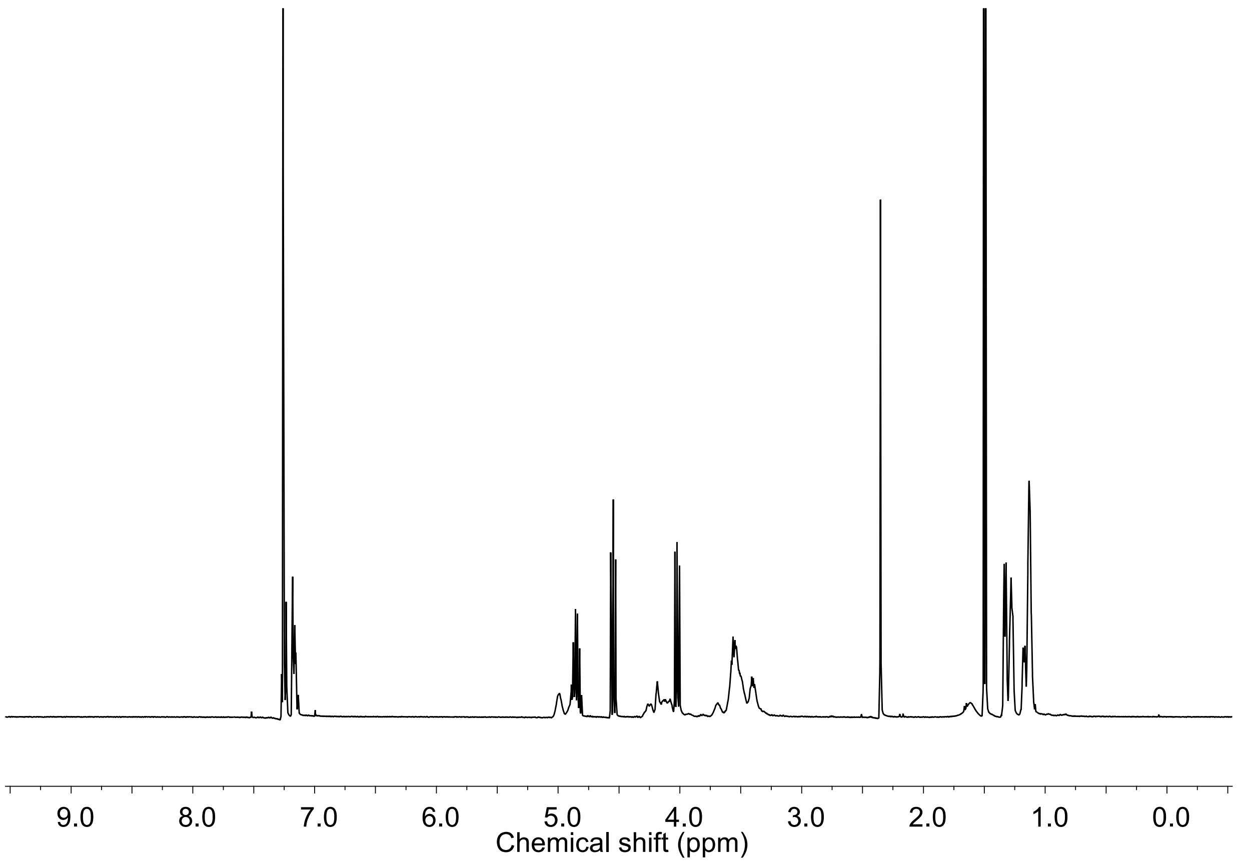
9.0 8.0 7.0 6.0 5.0 4.0 3.0 2.0 1.0 0.0
Chemical shift (ppm)

Fig. S30 ^{1}H NMR spectrum (400 MHz, $CDCl_3$) of the crude reaction mixture of the ROP of PO and CO_2 obtained by DMC-DEP. Polymerization Reaction condition: Catalyst amount = 50 mg , PO = 0.34 mol, PPG-600 = 0.25 mmol, toluene = 10 mL, P_{CO_2} = 30 bar, T_P = 105 °C, t_P = 3 h.

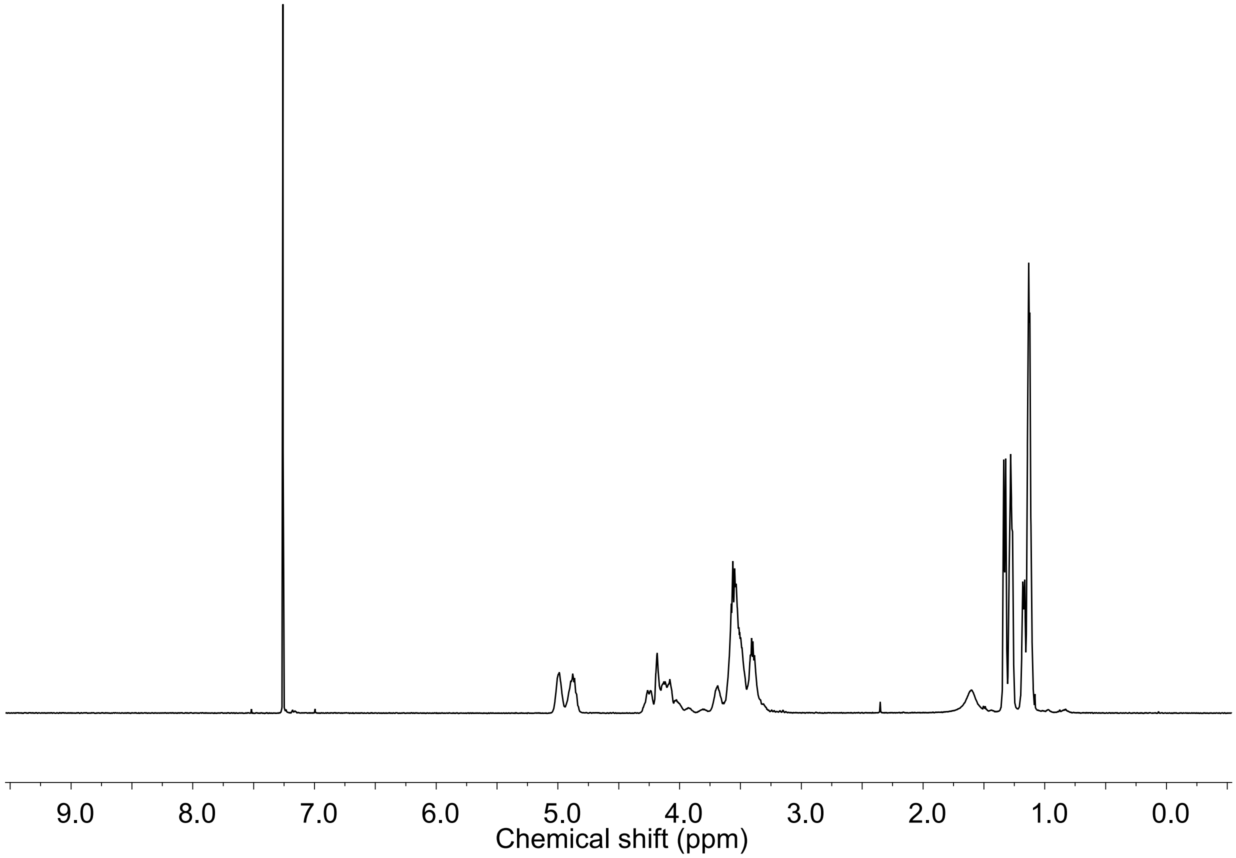

Fig. S31 ^{1}H NMR spectrum (400 MHz, $CDCl_3$) of the polycarbonate polyol obtained by DMC-DEP. Polymerization Reaction condition: Catalyst amount = 50 mg , PO = 0.34 mol, PPG-600 = 0.25 mmol, toluene = 10 mL, P_{CO_2} = 30 bar, T_P = 105 °C, t_P = 3 h.

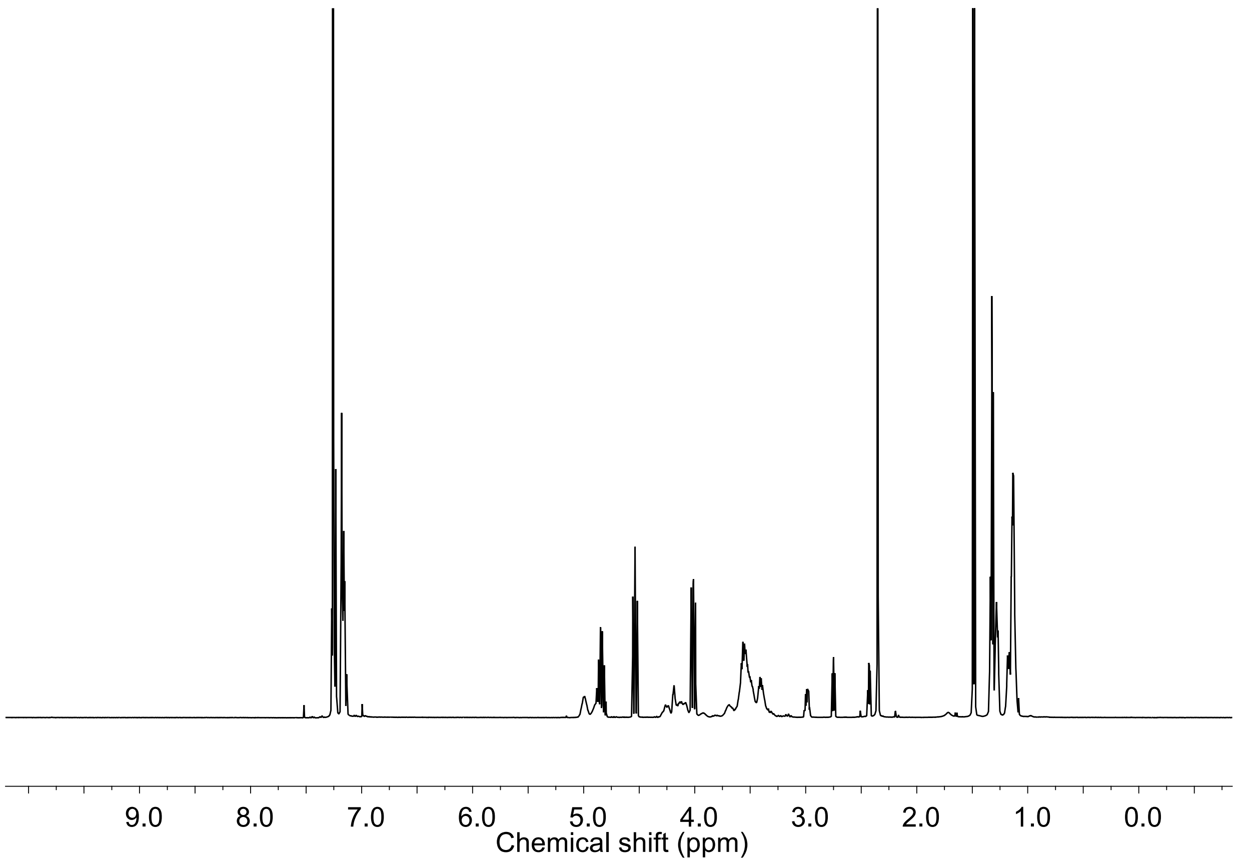

Fig. S32 ^{1}H NMR spectrum (400 MHz, $CDCl_3$) of the crude reaction mixture of the ROP of PO and CO_2 obtained by DMC-DEP. Polymerization Reaction condition: Catalyst amount = 50 mg , PO = 0.34 mol, PPG-600 = 2.5 mmol, toluene = 10 mL, P_{CO_2} = 30 bar, T_P = 105 °C, t_P = 3 h.

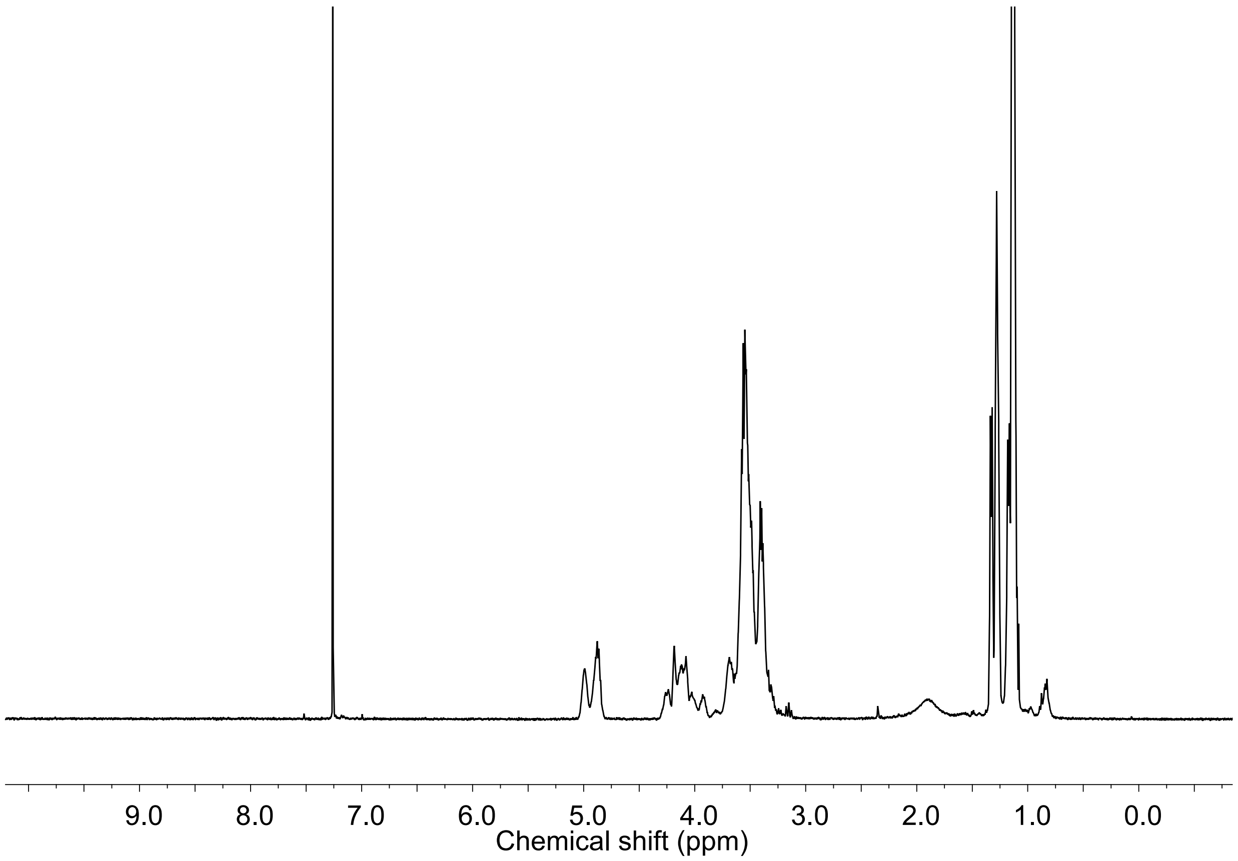

Fig. S33 ^{1}H NMR spectrum (400 MHz, $CDCl_3$) of the polycarbonate polyol obtained by DMC-DEP. Polymerization Reaction condition: Catalyst amount = 50 mg , PO = 0.34 mol, PPG-600 = 2.5 mmol, toluene = 10 mL, P_{CO_2} = 30 bar, T_P = 105 °C, t_P = 3 h.

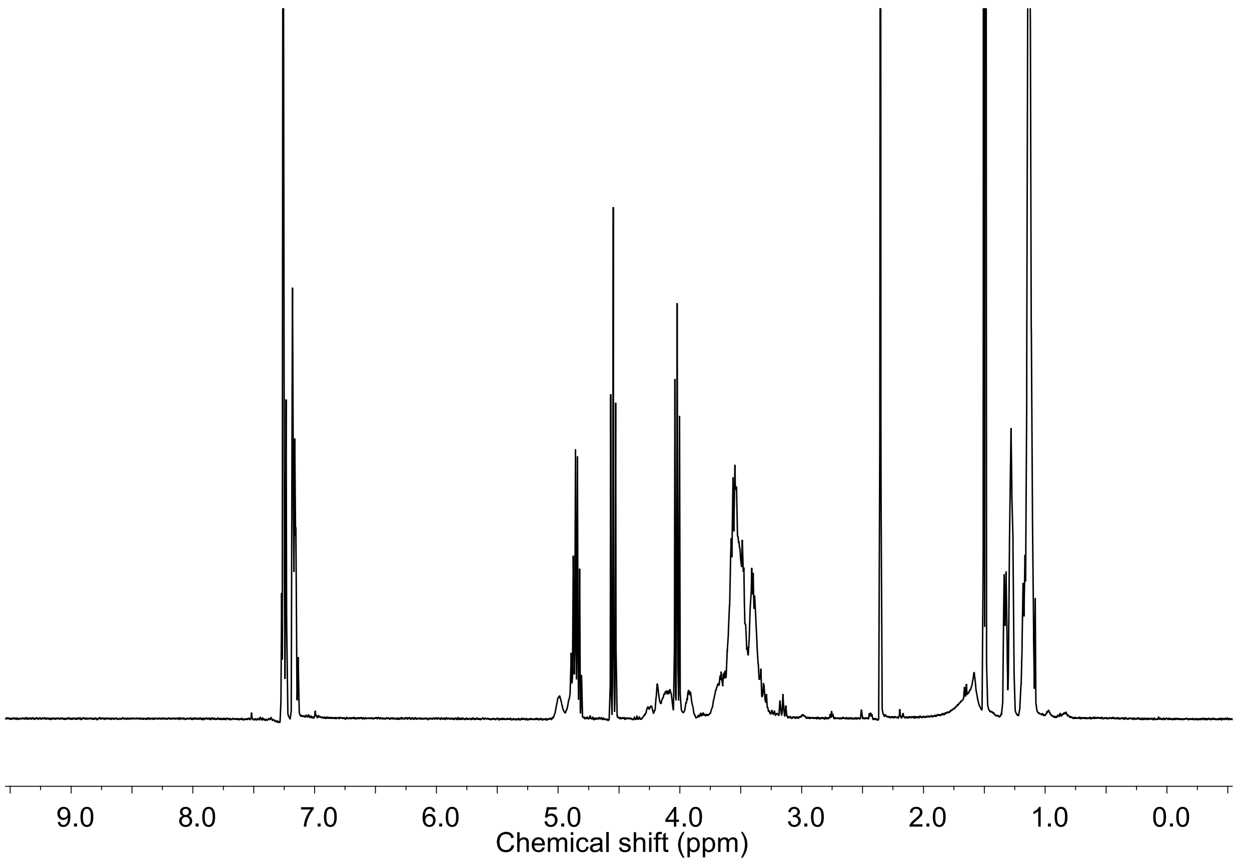

Fig. S34 ^{1}H NMR spectrum (400 MHz, $CDCl_3$) of the crude reaction mixture of the ROP of PO and CO_2 obtained by DMC-DEP. Polymerization Reaction condition: Catalyst amount = 50 mg , PO = 0.34 mol, PPG-600 = 12.5 mmol, toluene = 10 mL, P_{CO_2} = 30 bar, T_P = 105 °C, t_P = 3 h.

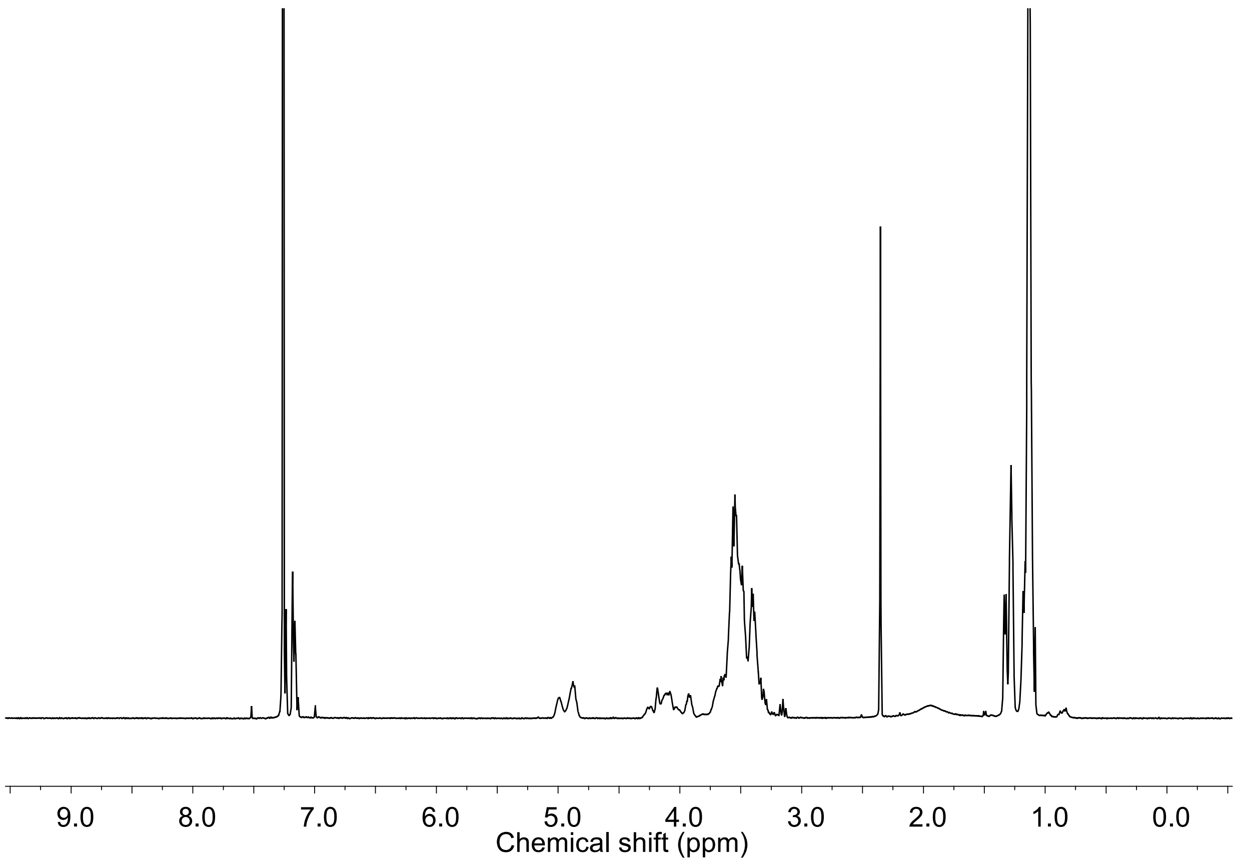

Fig. S35 ^{1}H NMR spectrum (400 MHz, $CDCl_3$) of the polycarbonate polyol obtained by DMC-DEP. Polymerization Reaction condition: : Catalyst amount = 50 mg , PO = 0.34 mol, PPG-600 = 12.5 mmol, toluene = 10 mL, P_{CO_2} = 30 bar, T_P = 105 °C, t_P = 3 h.

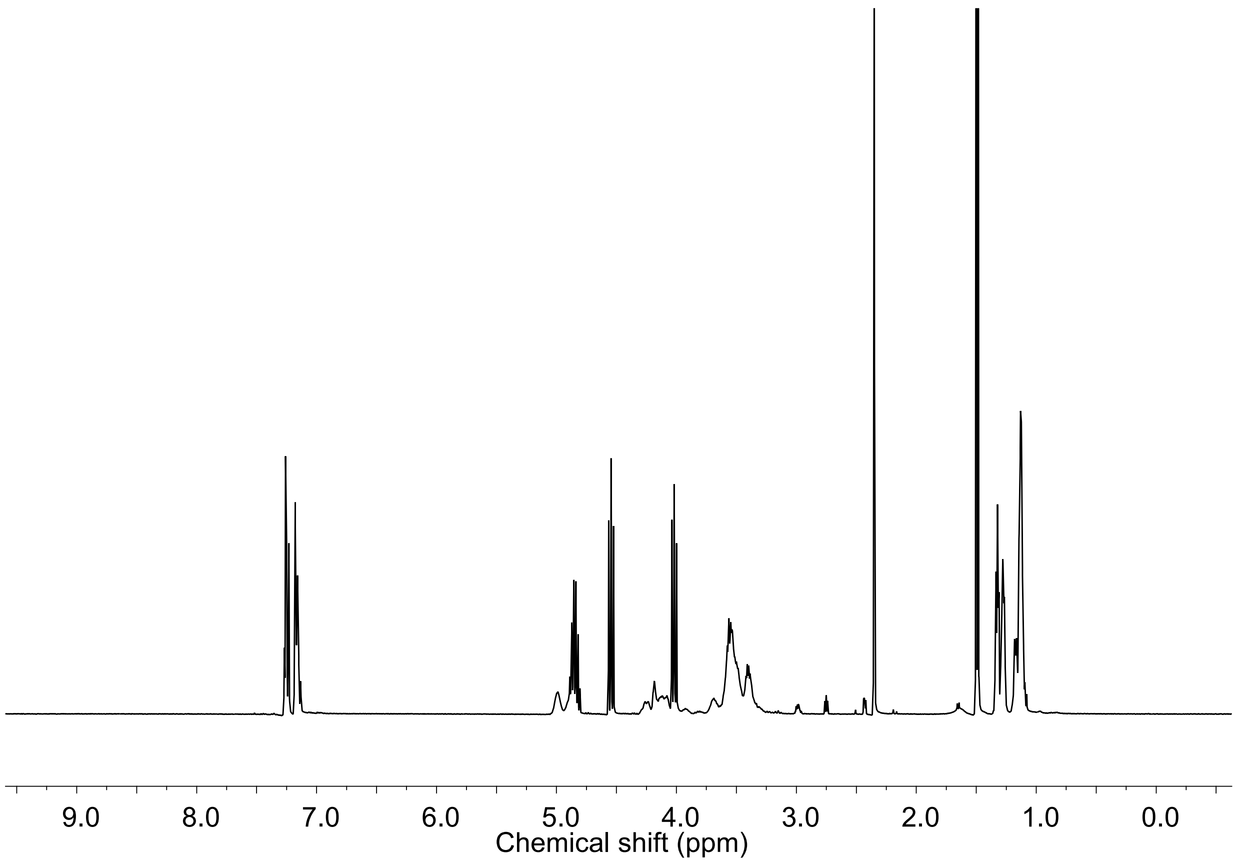

Fig. S36 ^{1}H NMR spectrum (400 MHz, $CDCl_3$) of the crude reaction mixture of the ROP of PO and CO_2 obtained by DMC-P(OEt)$_3$. Polymerization Reaction condition: Catalyst amount = 50 mg , PO = 0.34 mol, PPG-600 = 2.5 mmol, toluene = 10 mL, P_{CO_2} = 30 bar, T_P = 105 °C, t_P = 3 h.

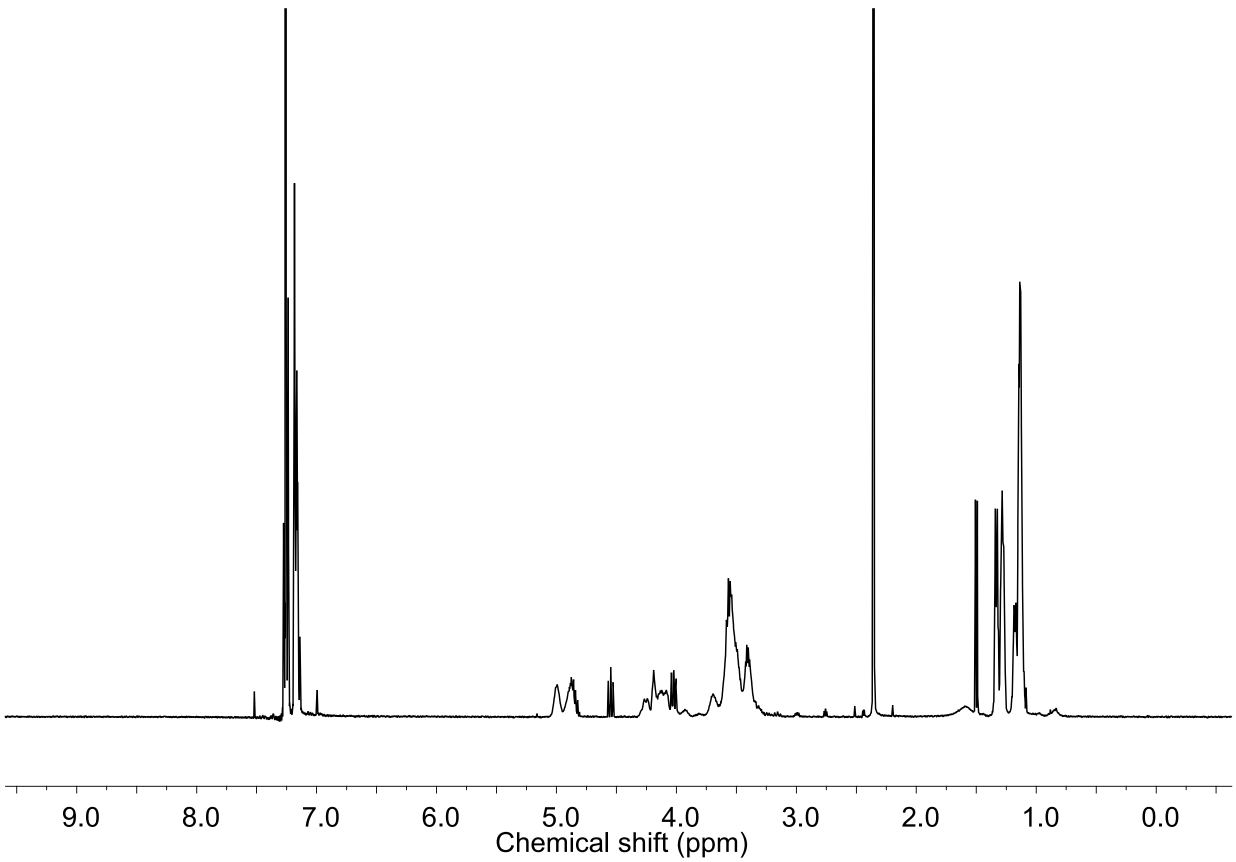

Fig. S37 ^{1}H NMR spectrum (400 MHz, $CDCl_3$) of the polycarbonate polyol obtained by DMC-$P(OEt)_3$. Polymerization Reaction condition: : Catalyst amount = 50 mg , PO = 0.34 mol, PPG-600 = 2.5 mmol, toluene = 10 mL, P_{CO_2} = 30 bar, T_P = 105 °C, t_P = 3 h.

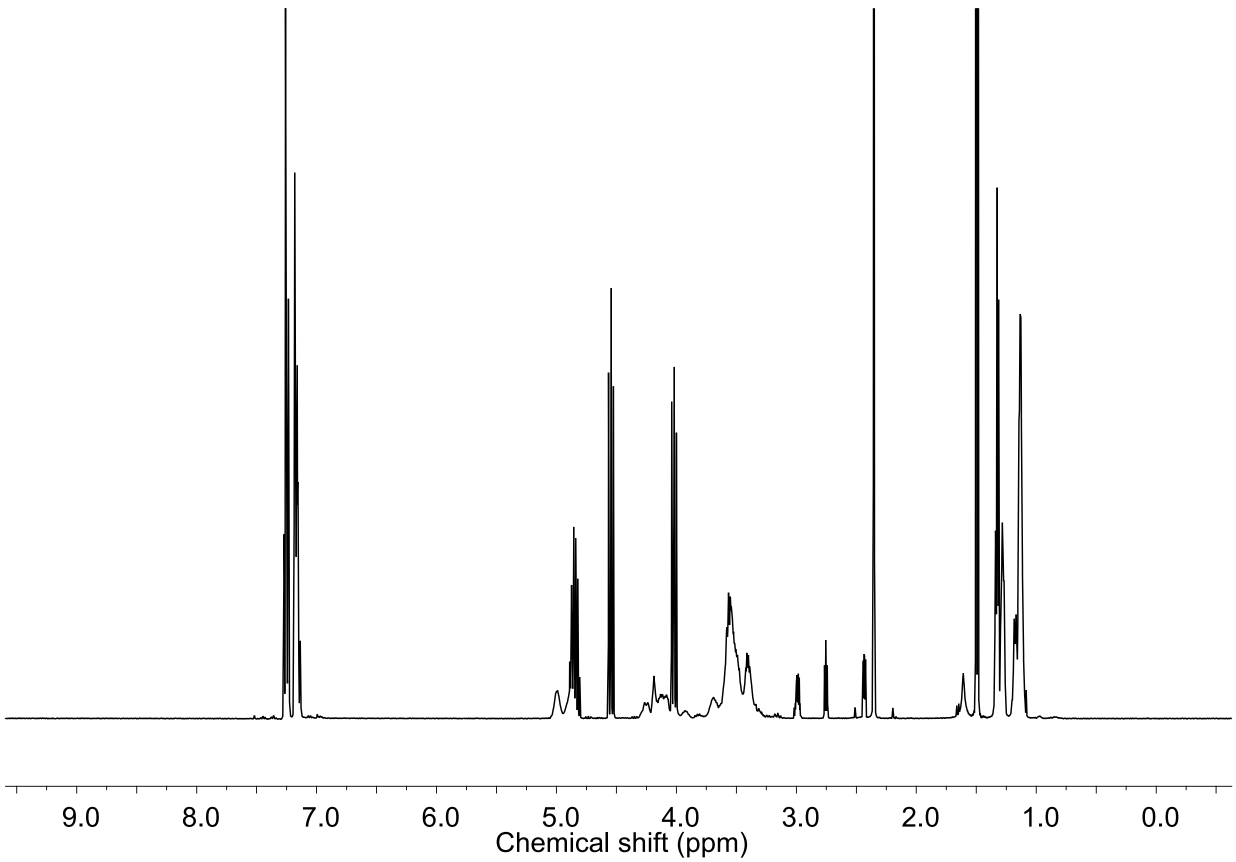

Fig. S38 ^{1}H NMR spectrum (400 MHz, $CDCl_3$) of the crude reaction mixture of the ROP of PO and CO_2 obtained by DMC-TEP. Polymerization Reaction condition: : Catalyst amount = 50 mg , PO = 0.34 mol, PPG-600 = 2.5 mmol, toluene = 10 mL, P_{CO_2} = 30 bar, T_P = 105 °C, t_P = 3 h

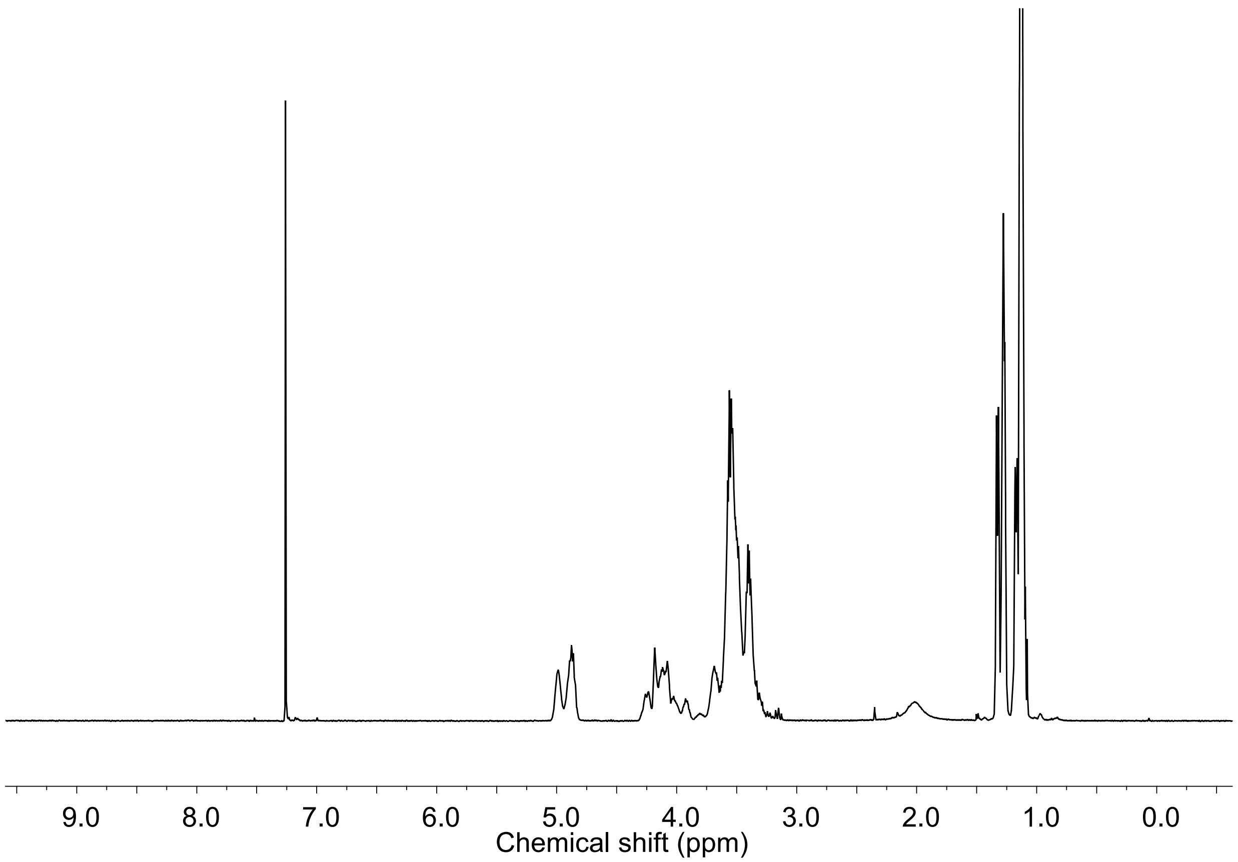

Fig. S39 ^{1}H NMR spectrum (400 MHz, $CDCl_3$) of the polycarbonate polyol obtained by DMC-TEP. Polymerization Reaction condition: : Catalyst amount = 50 mg , PO = 0.34 mol, PPG-600 = 2.5 mmol, toluene = 10 mL, P_{CO_2} = 30 bar, T_P = 105 °C, t_P = 3 h.

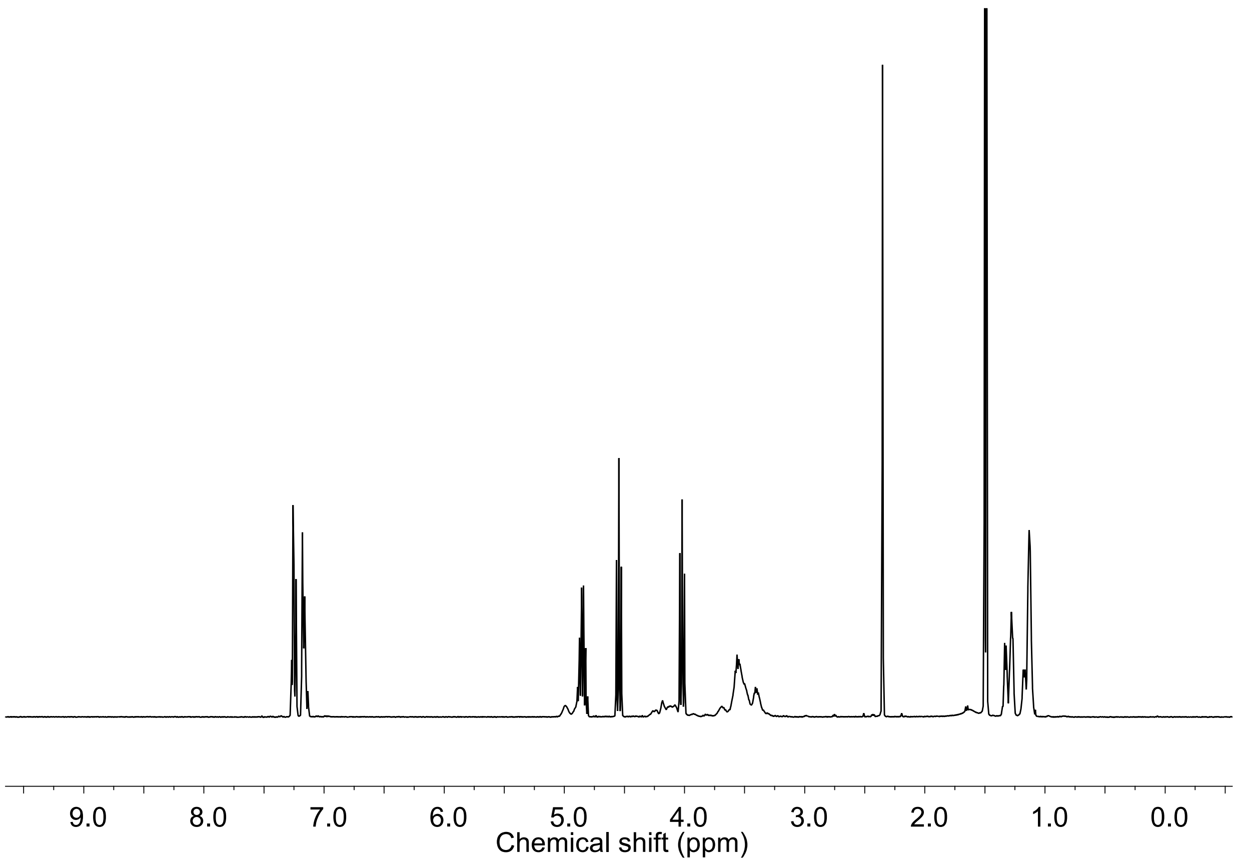

Fig. S40 ^{1}H NMR spectrum (400 MHz, $CDCl_3$) of the crude reaction mixture of the ROP of PO and CO_2 obtained by DMC-TEP without co-CA. Polymerization Reaction condition: : Catalyst amount = 50 mg , PO = 0.34 mol, PPG-600 = 2.5 mmol, toluene = 10 mL, P_{CO_2} = 30 bar, T_P = 105 °C, t_P = 3 h

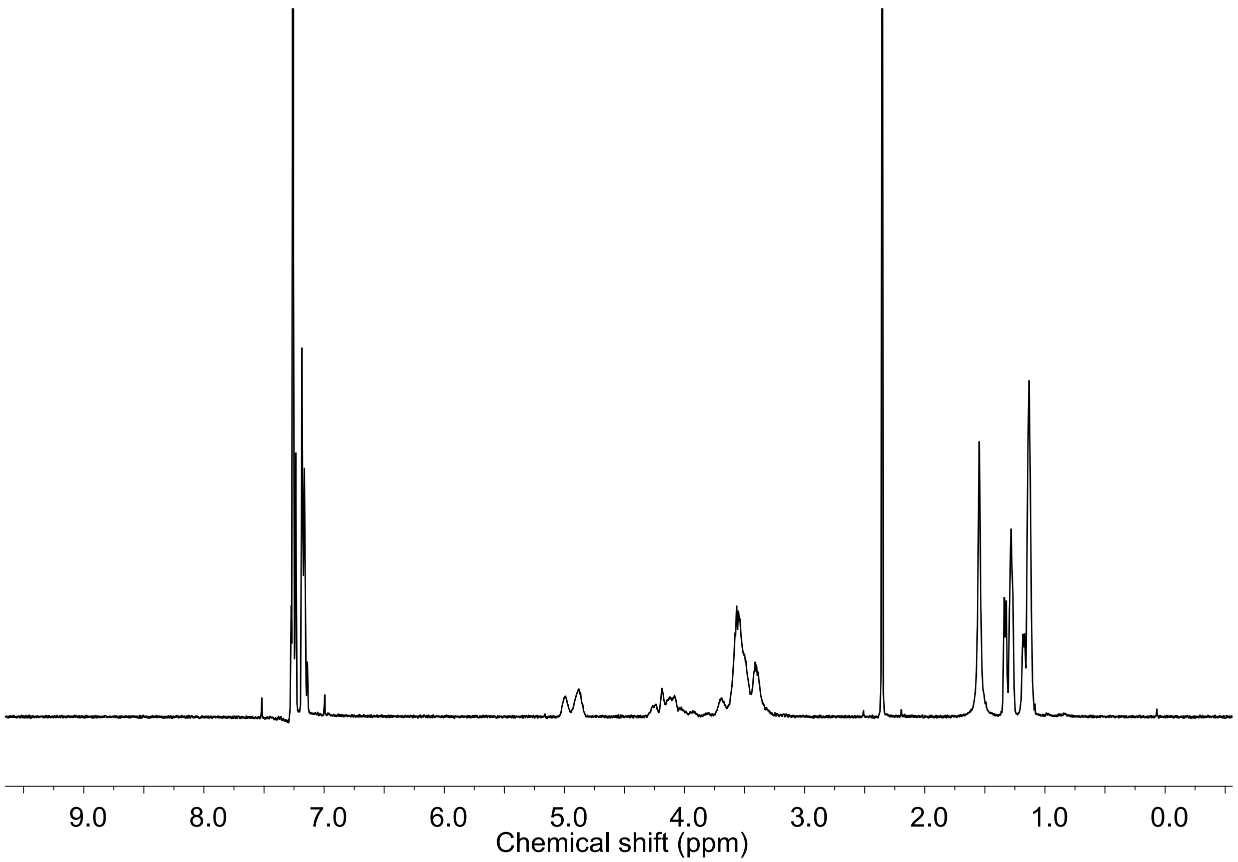

Fig. S41 ^{1}H NMR spectrum (400 MHz, $CDCl_3$) of the polycarbonate polyol obtained by DMC-TEP without co-CA. Polymerization Reaction condition: Catalyst amount = 50 mg , PO = 0.34 mol, PPG-600 = 2.5 mmol, toluene = 10 mL, P_{CO_2} = 30 bar, T_P = 105 °C, t_P = 3 h.

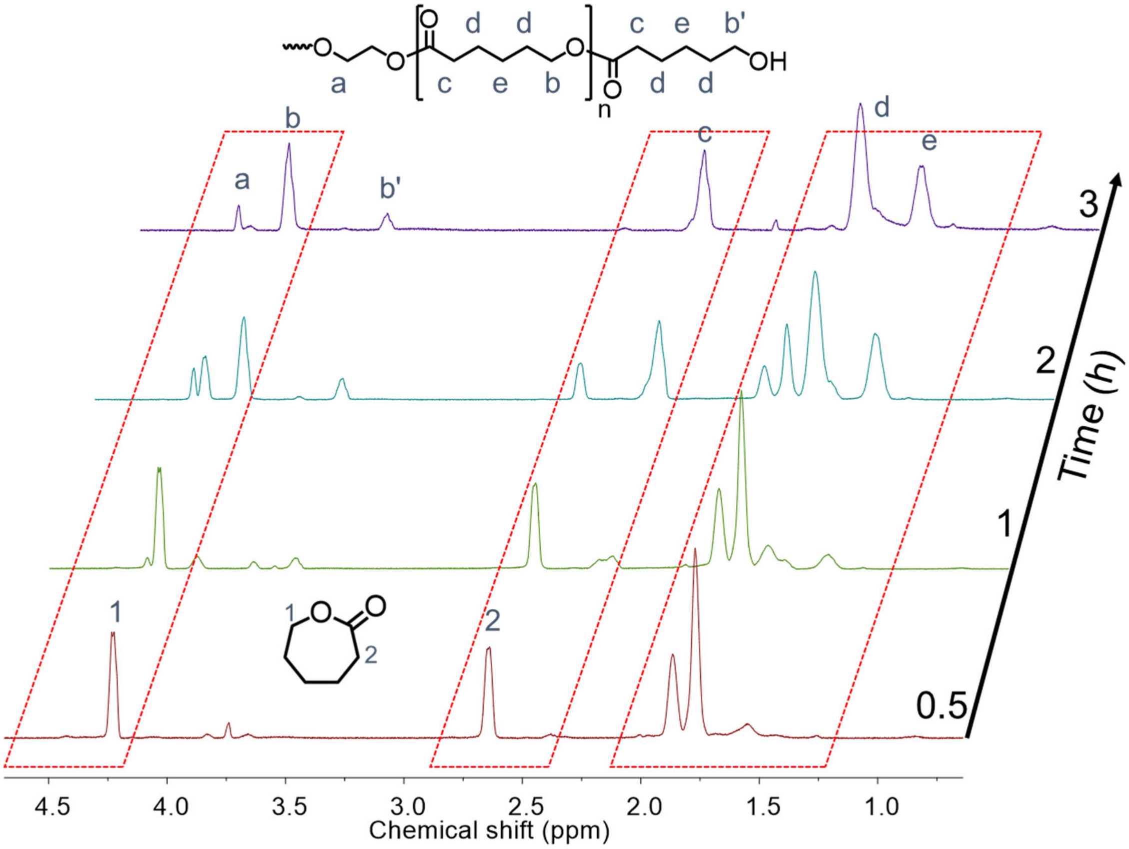

Fig. S42 ^{1}H NMR spectra (400 MHz, $CDCl_3$) of the crude reaction mixture of CL: polymerization using EG initiator and DMC-DEP catalyst. Reaction Conditions: catalyst amount = 10 mg ([Zn]0 = 30 mM), [CL]0 = 9 M, [CL]0/[EG]0 = 10, *Tp* = 160 °C.

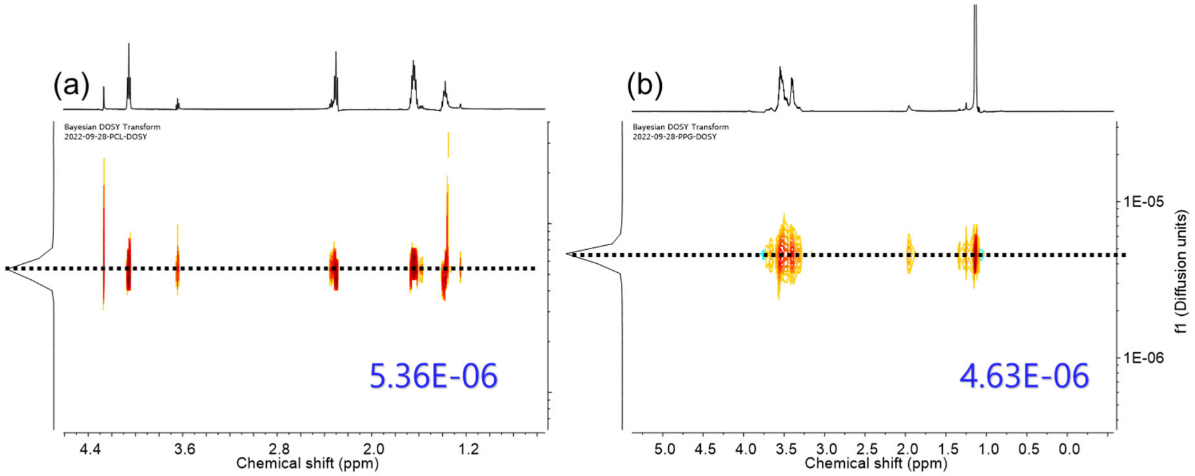

Fig. S43 2D DOSY NMR Spectra (600 MHz, $CDCl_3$) of (a) PCL 2000 and (b) PPG. Reaction condition: Catalyst amount = 50 mg, T_P = 115 °C.

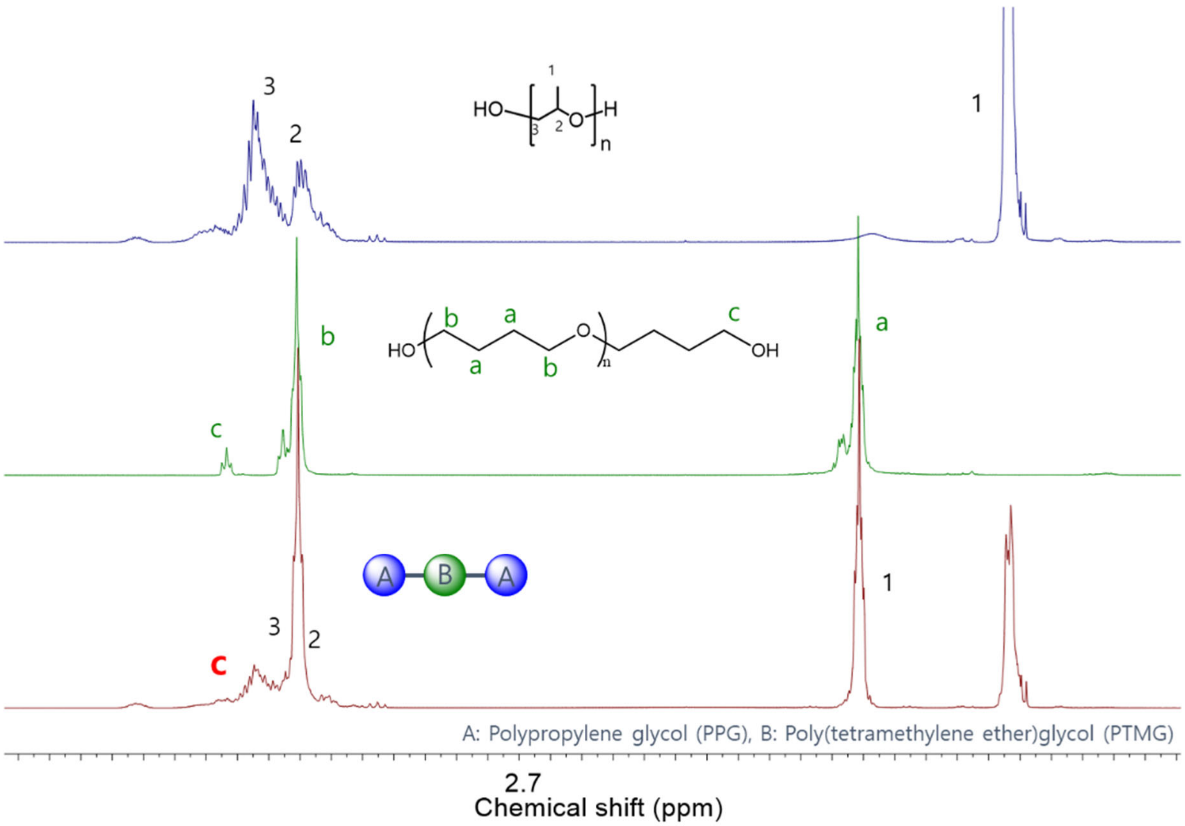

Fig. S44 [1] H-NMR spectra of PPG, PTMG and PPG-PTMG block copolymer (BCP-5).

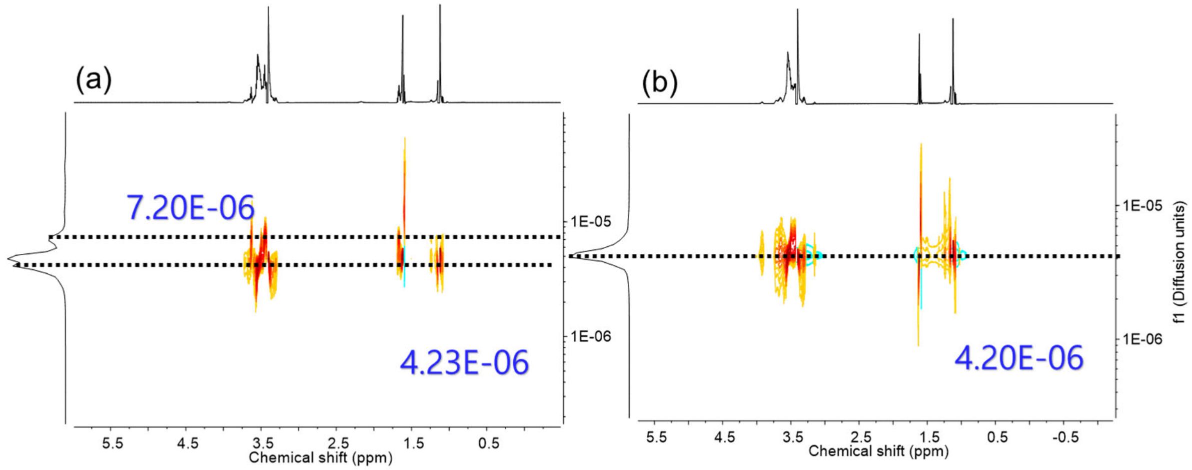

Fig. S45 2D DOSY NMR Spectra (600 MHz, $CDCl_3$) of (a) PPG and PTMG mixture and (b) BCP-5.

Reaction condition: Catalyst amount = 50 mg, T_P = 115 °C.

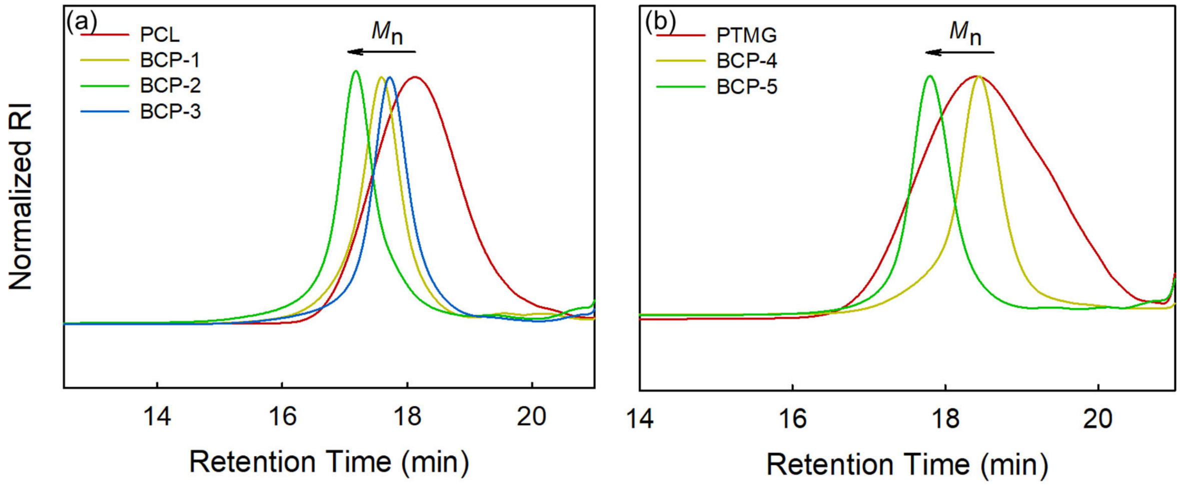

Fig. S46 GPC curves of the block copolymer obtained by batch ROP initiated by (a) PCL and (b) PTMG.

2. Supplementary tables

Table S1. DMC catalysts prepared using $ZnCl_2$(15 mmol), $K_3Co(CN)_6$ (1.5 mmol), and various type of OPC CAs.

Catalyst	Complexing agents						
	Type / Amount [a] (mmol)	Temperature [b] (°C)	M.W (g/mol)	Density (g/mL)	b.p. (°C)	Acidity (pKa)	Solubility in water (g/100 mL in 25 °C)
DMC-DMP	DMP/36	70	110	1.2	171	18.4	
DMC-DEP	DEP/26	30	138.1	1.072	188	13.0	miscible
DMC-DtBuP	DtBuP/16	70	193.2	0.960	225		
DMC-DMMP	DMMP/30		124.1	1.145	181	2.37	miscible
DMC- $P(OMe)_3$	$P(OMe)_3$/28	70	124.1	1.052	111		immiscible
DMC- $P(OEt)_3$	$P(OEt)_3$/19	70	166.2	0.969	156		immiscible
DMC-TMP	TMP/28	70	140.1	1.197	197		50
DMC-TEP	TEP/35	70	182.2	1.072	215	-9.1	miscible
DMC-TIP	TIP/26	70	224.2	0.970	224		immiscible
DMC-H_3PO_3	H_3PO_3/66	-	82.0	1.65	100	2.1/7.2/12.7	310

[a] Optimized amount through screening tests. [b] Catalysts and their optimized preparation temperature.

Table S2 Properties of triblock copolymers as co-complexing agent.

Type		PEG -PPG- PEG					PPG-PEG-PPG
Trade name (Pluronic), BASF		F-108	P-123	L-121	L-35	L-31	31R1
M_n		~14,600	~5,800	~4,400	~1,900	~1,100	~3,300
PEG (wt%)		82.5	30	30	50	10	10
PO units		50	69	67	16	16	2x26
EO units		2x132	2x19	2x13	2x11	2x2	8
Surface tension (dyn/cm); 25 °C, 0.1 wt. % in H_2O		41	34	33	49	47	34
Brookfield Viscosity (cP) at 25 °C		2,800	350	1,200	375	175	660
Cloud point (°C) at 1 wt. % aqueous solution		>100	90	14	73	37	25
T_m (°C)		60	39				
Softening point (°C)				5	7	−32	−25
Density (g/mL) at 25 °C			1.018	1.006	1.06	1.018	1.018
Hydrophilic-lipophilic balance (HLB)		27	8	1	19	5	1
pH (2.5% in H_2O)		6.0-7.4					
refractive index n20/D			1.465	1.454	1.461	1.453	1.454
Critical micelle concentration (%wt/v)	30 °C	0.8	0.005				
	45 °C	0.008					

[a] Boiling point.

Table S3 Summary of the FTIR results of DMC catalysts prepared by different OPC CAs

Catalyst	Vibration frequency (cm^{-1})						
	v(OH)	*v*(C≡N)	*δ*(H−O−H)	*v* (P=O)	*v*(C−O−C)	*v*(P-O)	*δ*(Co−CN)
DMC-pure	3650; 3424	2177	1612	−	−	−	450
DMC-tBuOH	3439	2194	1625	−	1086	−	474
DMC-DMP	3649; 3416	2192	1618	1198	1083	792	472
DMC-DEP	3414	2192	1616	1237	1069	788	468
DMC-D*t*BuP	3444	2199	1617	1259	1086		475
DMC-DMMP	3414	2189	1618	1233	1071	796	468
DMC-$P(OMe)_3$	3419	2193	1619	1258	1077	862	473
DMC- $P(OEt)_3$	3411	2197	1619	1231	1070	789	474
DMC-TMP	3416	2191	1620	1222	1059	850	473
DMC-TEP	3419	2189	1620	1249	1074	800	471
DMC-TIP	3411	2186	1617	1221	1042	785	468
DMC-H_3PO_3							

Table S4 Summary of the FTIR results of optimized DMC catalysts prepared by different temperature

Catalyst [a]	T(°C)	Vibration frequency (cm^{-1})			
		v(C≡N)	v (P=O)	v(P-O)	δ(Co−CN)
DEP	−	−	1254	770	−
DMC-DEP	30	2192	1237	788	468
	50		1220		471
	70		1213		474
	90		1232		474
DMC-H_3PO_3	30	2181		792	452
$P(OEt)_3$	−	−	−	720	−
DMC- $P(OEt)_3$	30	2195	−		465
	50	2195	1230		470
	70	2197	1231	789	474
	90	2196	1225		472

Table S5 Summary of the XPS results of DMC catalysts prepared by different CAs

Catalyst (prep. temp. in ℃) [a]	Zn 2p3		Co 2p3		O 1s		N 1s		C 1s		Cl 2p		P 2p	
	BE (eV)	[AT] (%)	BE (eV)	[AT] (%)	BE (eV)	[AT] (%)	BE (eV)	[AT] (%)	BE (eV)	[AT] (%)	BE (eV)	[AT] (%)	BE (eV)	[AT] (%)
$ZnCl_2$	1023.7	–	–	–	–	–	–	–	–	–	–	–	–	–
$K_3Co(CN)_6$	–	–	781	–	–	–	–	–	–	–	–	–	–	–
DMC-pure	1021.9	–	781.7	–	531.2		398.2	–	285	–	198.5	–	–	–
DMC-DEP (30)	1023.2	4.8	783.1	2.7	533.4	15	399.4	12.3	286.6	61.9	199	2.2	134.4	1.1
DMC-DEP (50)	1023.0	6.5	783.3	3.4	533.0	22.2	399.6	14.2	286.6	48.7	199	1	134.2	3.9
DMC-DEP (70)	1022.6	5.9	782.8	3.5	532.8	20.5	399.1	15.7	286.3	50.8	198.4	0.8	133.9	2.9
DMC-DEP (90)	1022.8	1.5	783.1	0.8	533.3	18	399.3	4.2	285.1	74.3	198.6	0.2	133.9	1
DMC-$P(OEt)_3$ (30)	1022.9	0.9	782.9	0.6	533.2	17.6	399.3	2.5	285.1	77.7	198.6	0.4	134.5	0.3
DMC-$P(OEt)_3$ (50)	1023.4	6.6	783.1	3.9	533.2	19.3	399.6	16	286.5	50.5	199.0	0.8	134.3	2.9
DMC-$P(OEt)_3$ (70)	1023.2	2.4	782.9	1.5	533.3	16.8	399.4	6.4	285	71.1	198.9	1.1	134.6	0.6
DMC-$P(OEt)_3$ (90)	1022.9	2.5	782.9	1.4	533.1	19.1	399.5	6.4	285.1	69	198.7	0.2	133.9	1.4
DMC-TEP	1023.1	2.6	782.7	1.4	533.5	15.8	399.6	6.4	285	71.5	199.7	1.1	134.9	1.2
DMC-DMMP	1022.6	2.7	782.6	1.9	532.9	17.6	399.1	9.6	286.4	66.4	198.4	0.5	133.9	1.4

[a] Catalysts and their optimized preparation temperature.

Table S6 Results for the semi-batch ROP of PO using various DMC catalysts

Entry	Catalyst	Preparation condition			TOF (min^{-1})	Polyol properties			
		CA	*V*(mL) [a]	T (°C)		M_n [b] ($g\ mol^{-1}$)	*Đ* [c]	unsat. ($meq\ g^{-1}$) [d]	*F* [e]
1	DMC-DMP	DMP	0.1	70	215	5600	1.15	0.00765	1.89
2	DMC-DEP	DEP	0.1	30	726	4300	1.15	0.00793	1.88
3	DMC-DEP	DEP	0.1	50	404	–	–	–	
4	DMC-DEP	DEP	0.5	50	318	–	–	–	
5	DMC-DEP	DEP	1.0	50	47	–	–	–	
6	DMC-DEP	DEP	0.1	70	360	–	–	–	
7	DMC-DEP	DEP	0.1	90	56	–	–	–	
8	DMC-D*t*BuP	D*t*BuP	0.1	70	302	4700	1.12	0.00850	1.98
9	DMC-DMMP	DMMP	0.1	30	–	–	–	–	
10	DMC-DMMP	DMMP	0.1	50	–	–	–	–	
11	DMC-DMMP	DMMP	0.1	70	–	–	–	–	
12	DMC-$P(OMe)_3$	$P(OMe)_3$	0.1	70	274	4600	1.14	0.00623	1.92
13	DMC- $P(OEt)_3$	$P(OEt)_3$	0.1	30	334	–	–	–	
14	DMC- P(OEt)3	$P(OEt)_3$	0.1	50	253	–	–	–	
15	DMC- P(OEt)3	$P(OEt)_3$	0.5	50	131	–	–	–	
16	DMC- P(OEt)3	$P(OEt)_3$	1.0	50	154	–	–	–	

17	DMC- P(OEt)3	$P(OEt)_3$	0.1	70	405	4400	1.17	0.00850	2.00
18	DMC- P(OEt)3	$P(OEt)_3$	0.1	90	57	–	–	–	
19	DMC-TMP	TMP	0.1	70	15	–	–	–	
20	DMC-TEP	TEP	1.0	30	557	–	–	–	
21	DMC-TEP	TEP	0.1	50	191	–	–	–	
22	DMC-TEP	TEP	0.5	50	486	–	–	–	
23	DMC-TEP	TEP	1.0	50	550	–	–	–	
24	DMC-TEP	TEP	1.0	70	574	4100	1.14	0.00595	1.95
25	DMC-TEP	TEP	1.0	90	560	–	–	–	
26	DMC-TIP	TIP	1.0	70	403	4300	1.16	0.00623	1.98
27	DMC-H_3PO_3	H_3PO_3	0.1	30	51	–	–	–	
28 [f]	DMC-*t*BuOH	*t*BuOH	60 mmol	50	212	3200	1.12	0.0065	1.72
29 [f]	DMC-*t*BuOH	*t*BuOH	120 mmol	50	174	–	–	–	
30 [f]	DMC-*t*BuOH	*t*BuOH	150 mmol	50	154	–	–	–	

[a] Amount of OPCs. [b] Number average molecular weight measured by GPC (THF solvent). [c] Polydispersity index. [d] Unsaturation level. [e] Functionality determined by titration. [f] Obtained from ref [1]. Reaction condition: Catalyst loading (n_{Zn}) = 0.3 mmol, PO = 3.5 mol, PPG-600 = 50 mmol, T_P = 115 °C.

3. References

[1] C.H. Tran, S.J. Lee, B.-r. Moon, E.-g. Lee, H.-k. Choi, I. Kim, Organonitriles as complexing agents for the double metal cyanide-catalyzed synthesis of polyether, polyester, and polycarbonate polyols, Catal. Today, 418 (2023) 114125.

[2] R.G. Pearson, Absolute electronegativity and hardness: application to inorganic chemistry, Inorg. Chem., 27 (1988) 734−740.
